# Social dynamics signals in archaeological time-series: How cultural transmission and production biases shape assemblage diversity

**DOI:** 10.1371/journal.pone.0358135

**Published:** 2026-10-07

**Authors:** Aleksandr Diachenko, Detlef Gronenborn

**Affiliations:** 1 Institute of Archaeology, National Academy of Sciences of Ukraine, Kyiv, Ukraine; 2 Leibniz-Zentrum für Archäologie, Mainz, Germany; 3 Johannes Gutenberg University, Mainz, Germany; University of Michigan, UNITED STATES OF AMERICA

## Abstract

High-resolution studies on scales of decades to centuries indicate that social systems shift to greater complexity and back, a phenomenon usually referred to as social cycles. In archaeological data, such cycles are reflected in the increase and decrease of diversity in material culture signals. However, the exact correlation between assemblage diversity cycles and social complexity remains unclear. Addressing this topic, our model investigates the impact of cultural transmission and production biases on the variation in material culture sets and explains how the observable variability in empirical evidence results from the same basic complex dynamic behavior. A single mathematical algorithm describes path-dependency, gradual changes, and rapid transformations of the empirically visible assemblage composition as the replacement of components of material culture in archaeological assemblages. Our simulations capture the direct impact of short-term fluctuations and delayed sensitive dependence on the initial conditions (chaotic effects) of the assemblage diversity. The model finds a decrease in variation (standardization) in assemblages to be an outcome of production bias, potentially reflecting changes in social complexity.

## Introduction

One of the major questions not only in archaeology and history but also in political anthropology, sociology, and political science is: why do social systems change from simple to more complex stages and back? While on long time scales – i.e., millennia – social proxies seem to follow a trajectory of continuously increasing complexity, culminating in the Anthropocene and the Grand Acceleration phenomenon [1,2], high-resolution studies on shorter time-scales – i.e., decades to centuries – indicate that systems regularly shift to greater complexity and then revert, a phenomenon usually referred to as social cycles [3,4]. Occasionally, social cycle trajectories may be interrupted by scale shifts [4–6]. In archaeological datasets, such social cycles and scale shifts may be visible in diversity changes in material culture (Fig 1a).

**Fig 1:**
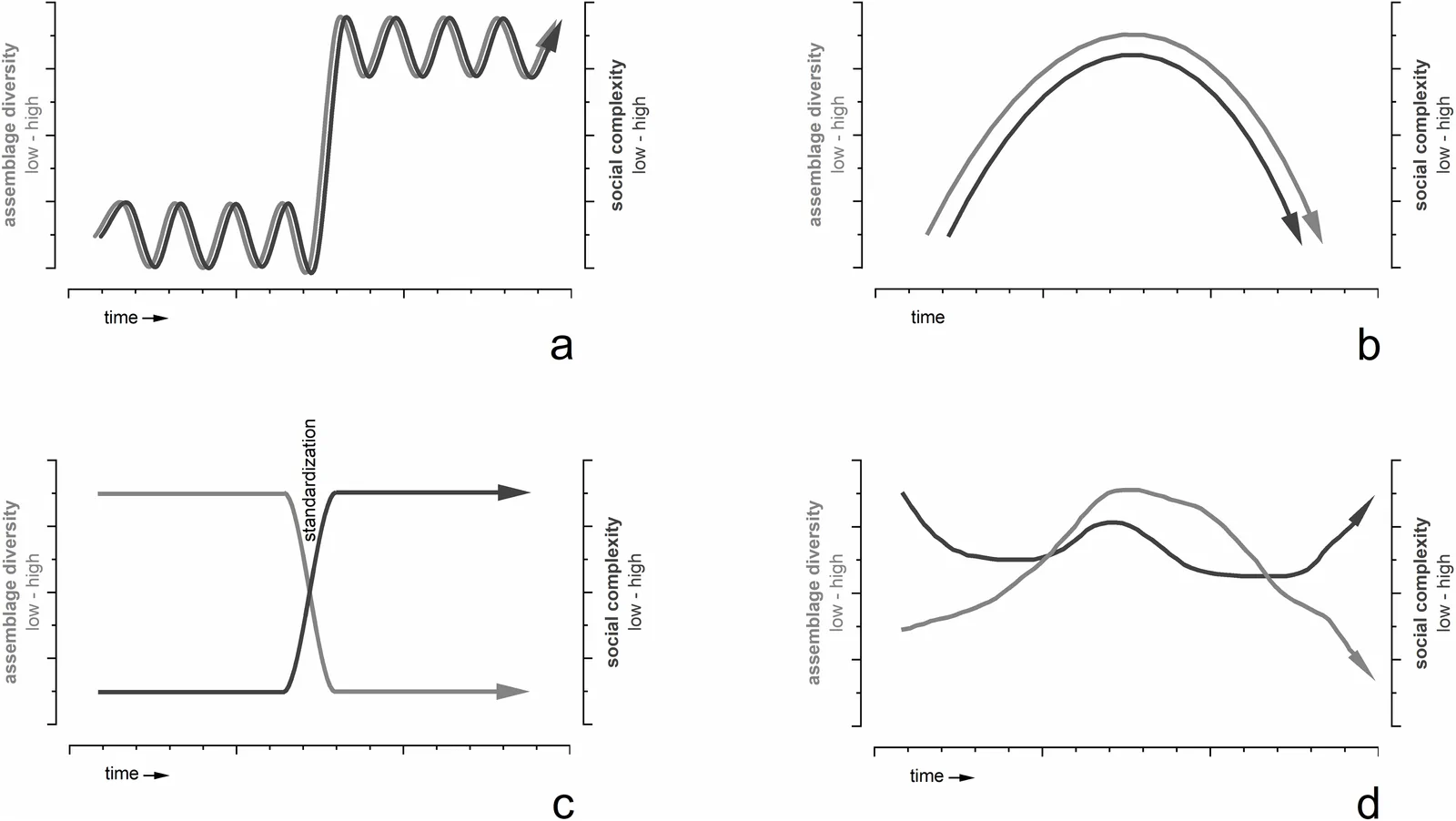
Social complexity and assemblage diversity. a – Cyclical behavior of material culture diversity signals and social complexity with hypothetical scale shift as a basic template for cultural evolution, b – positively correlated assemblage diversity and social complexity trajectories (boom-bust cycle), c – negatively correlated assemblage diversity and social complexity trajectories (standardization), d – unrelated assemblage diversity and social complexity trajectories.

Significant inroads into the theory and practice of studying assemblage diversity in our discipline are demonstrated by volumes edited by Leonard & Jones and Eren & Buchanan [7,8], as well as in various journal articles [9–18]. However, understanding archaeological diversity patterns remains a major challenge [19–21]. Our paper contributes to this topic by investigating possible interrelations between changes in social complexity and assemblage diversity through the analysis of production biases (see below).

The available evidence, representing both positive and negative correlations between the diversity of archaeological assemblages and social entities, might look controversial, as we demonstrated by the examples from the European Neolithic and Eneolithic assemblages [22]. More specifically, based on a time series of ceramics, Gronenborn et al. inferred a coarse correlation between population density and social dynamics for the western Central European Early Neolithic Linear Pottery culture (Germ. Linearbandkeramik – LBK) [4,23–25]. Based on the informed assumption that the LBK decoration system coded social content, a succession of social phases was deduced from changes in the stylistic diversity of pottery decoration motifs. The multilayered LBK pottery decoration system revealed two interrelated cycles (e.g., Fig 1b, also see below): an overarching cycle, formed by decoration techniques of major motifs (band types), represents a more general western LBK meta-identity, while the sub-cycles, based on changes in secondary decoration motifs, represent changes in the self-identification of and between social sub-groups [25]. A similar case for another region of the western LBK based on similar datasets was made by Peters and Zimmermann [26], but linked to the more aggregate concept of social resilience. In this context, it is important to note that population size and density potentially impact both the diversity of social entities [27] and archaeological assemblages [13,28–32]. Therefore, a positive correlation between social complexity and assemblage diversity cycles (Fig 1b) may be driven by boom-and-bust demographic dynamics [22,33–38].

Other studies reveal a negative correlation between social complexity and assemblage diversity (Fig 1c). These approaches treat standardization, defined as a decrease in variation, as evidence of an increase in social complexity [39–42]. The standardization hypothesis states that the uniformity of artifacts increases due to increasing production rates correlated with economic specialization (see [41] and references therein). Citing [43], Roux emphasizes that intentional (controlled by a producer) and mechanical (unintentionally introduced and related to technology) factors, resulting from the sophistication of the production process, push the decrease in variation [41].

Lastly, besides positive and negative correlations between assemblage diversity and social complexity, there might be no correlation between the two at all (Fig 1d): According to Robert Dunnell, evolutionary archaeology distinguishes between style and function [44]. While style denotes cultural traits lacking detectable selective value, function refers to the “traits that directly affect the Darwinian fitness of the populations in which they occur” [44: 199]. The diversity of stylistic traits in an assemblage are caused by cultural drift (random events) and ‘mutation’ (local innovation or adoption of cultural traits from other groups), both of which shape neutral cultural transmission [14,45–48]. Therefore, changes in assemblage composition may result from transmission biases which are not related to social transformations (for instance, frequency-dependent biases: e.g., [49–52]). As exemplified by the case of the Western Trypillia (also known as Western Tripolye) culture (WTC) pottery in the Southern Bug and Dnieper interfluve in present-day Ukraine (see below), the replacement of several forms or styles along with the introduction and extinction of several vessel types is visible as a rise-and-fall pattern in assemblage diversity c. 4100−3600 BCE [10]. Although this pattern looks similar to the stylistic diversity cycles in the LBK assemblages, it does not signal social transformation.

At first glance, the above-cited examples may create the impression that any relationship between social complexity and assemblage diversity is distinct and heavily context-dependent [22]. Therefore, a formal description of such distinct relationships would require different models. However, we argue here that the observable variability in the empirical evidence results from the same underlying dynamic behavior and may be described by a single mathematical algorithm. The presented model scales social complexity to production and consumption, and investigates the synergetic impact of cultural transmission and production biases on variations in material culture sets. However, our analysis of the impact of threshold parameters on the model behavior leads us to caution in clearly distinguishing social dynamics signals from other factors determining curve progressions in archaeological data.

## Model description

We begin with a brief introduction to social complexity and the most recent approaches in quantifying assemblage diversity. We then present the basic assumptions and explain how they are accounted for by the mathematical component of our model.

### Defining social complexity

Social complexity in archaeology is generally considered within a wider framework of complexity theory [53,54]. Complex systems may be defined as “large networks of components with no central control and simple rules of operation giv[ing] rise to complex collective behavior, sophisticated information processing, and adaptation via learning and evolution” [55] (cited from [56: 93]). Emergent properties resulting from the interaction of component parts arise from differences in activities, aims, decisions, etc. of individuals and groups [53–58]. From this, it follows that changes in material culture diversity are inherently linked to social complexity changes.

Cultural traits are inscribed into objects made by people, for people [59,60]. Therefore, this study breaks down social complexity into a producer-consumer relationship and considers that producers are most frequently also the consumers of their own products, especially in small-scale societies. Unlike a cultural trait forced into a certain taxonomic unit (see below), an individual may participate in several groups of consumers and producers simultaneously. The latter is somewhat captured by the categorization of craft organization, e.g., part-time vs. full-time [41,42]. Here, we limit our model to stylistic variation, assuming only quantitative changes in consumer-producer relationships. For the sake of simplicity, the model considers only a single category of the produced and consumed objects, leaving the aggregation of the overall production and consumption as the combined signal of social complexity for future research.

### Estimating assemblage diversity

Material culture assemblages are the aggregates of sub-assemblages, sub-sub-assemblages, etc., combining spatially and chronologically bounded sets such as artifacts, features, and sites [20]. Originating from information theory and widely applied in various fields of science, diversity measures are the aggregates of variation in assemblages. This aggregation is often quantitatively approached through the estimation of gamma diversity borrowed from ecology. Gamma diversity integrates alpha diversity, which is the diversity of individual assemblages, and beta diversity, representing a magnitude of sample variation [10,20,61–65]. This study avoids the issue of estimating beta diversity by introducing a simplification into the model: samples are considered homogeneous. In other words, we set beta diversity to 1, gamma diversity equals alpha diversity, and we describe our hypothetical assemblage as being composed of identical sub-assemblages.

Archaeological research mostly deals with entropy as the diversity index. More recent approaches suggest shifting from diversity indices to diversity magnitudes (hereinafter – ‘diversity’), which are exponentiated entropies [10,63,64]. For instance, archaeological applications frequently consider Shannon entropy (‘heterogeneity’: [66]), whereas depending on the sample properties and aims of studies, the most popular diversities are the ‘richness’ (number of units) and ‘evenness’ (a ratio of exponentiated ‘heterogeneity’ to ‘richness’: e.g., [17,18,67]). Although graphs representing change in diversity and entropy show identical shapes, the application of the diversity magnitude could make more sense as it provides a number of the so-called ‘effective’ units [10,63].

We estimate the Shannon diversity (Equation 2) as the exponentiated Shannon entropy for the units belonging to the same level of taxonomic hierarchy. The Shannon entropy [66] is expressed as follows:


$$\begin{array}{c} H=-\sum_{i}p_{i}\;\text{ln}\;p_{i} \end{array}$$ (1)

where $p_{i}$ is the proportion *i*-th trait ($\sum p_{i}=\text{1}$),

Respectively, Shannon diversity is estimated as follows:


$$\begin{array}{c} D=\text{exp}\left(-\sum_{i}p_{i}\;\text{ln}\;p_{i}\right) \end{array}$$ (2)

As seen from Equations 1 and 2, data categorization has an essential impact on estimations because diversity magnitudes are dependent on the number of units and the proportion of traits attributed to these units (for the detailed discussion, see: [8,20,63]).

### Modeling socio-cultural cycles

Lastly, we conceptualize socio-cultural cycles as rise-and-fall patterns in assemblage diversity. The basic assumptions of our approach are summarized as follows:

1) Cultural ‘mutations’ and transmission biases operate within a framework of consumer-production relationships. As such, production biases impact the magnitudes of thresholds constraining the proportion of traits.
2) Total production and consumption are correlated with population size.
3) Taxonomic levels of traits have to be considered as diversity of traits increases top–down the taxonomic hierarchy [68].

In the long run, producer-consumer ratios (social complexity) cause changes in production intensity and, therefore, changes in assemblage diversities even given all other factors being equal (Table 1). Unless the total consumption and the number of producers change at the same rate over the chronological meso-scale (Table 1), the synergetic effect of production biases (resulting from changing consumer-producer ratios) and cultural transmission biases on assemblage diversity is highly likely. The more production and consumption shift away from self-sustaining (items are produced and consumed individually; the distribution of styles only results from cultural transmission mechanisms), the more complicated it gets to distinguish between production and transmission biases.

**Table 1:** Producer-consumer relationships and production intensity as a marker of social complexity.

Total production and consumption	Number of producers	Production intensity as a marker of social complexity
**Increases**	Decreases	Increases
	Remains stable	Increases
	Increases at the lower rate than the number of consumers	Increases
	Increases at the same rate as the number of consumers	Remains stable
	Increases at the higher rate than the number of consumers	Decreases
**Remains stable**	Decreases	Increases
	Remains stable	Remains stable
	Increases	Decreases
**Decreases**	Decreases at the higher rate than the number of consumers	Increases
	Decreases at the same rate as the number of consumers	Remains stable
	Decreases at the lower rate than the number of consumers	Decreases
	Remains stable	Decreases
	Increases	Decreases

Considering the taxonomic structure of archaeological data, this study modifies the approach proposed by Diachenko & Sobkowiak-Tabaka by distinguishing between the primary and secondary units, which combine cultural traits of the same taxonomic level [69]. Primary units dominate by their proportion over secondary units in sets *m* of the sa*m*e taxonomic level (λ) at the initial chronological stage. As the proportion of the primary unit starts to decrease, this primary unit shifts to a secondary one. Change in the proportion of traits over time is expressed as follows [69]:


$$\begin{array}{c} \sum \left(p_{{{\alpha_{m}}_{\lambda }}_{t}}\frac{{r_{\alpha }}_{m_{\lambda }}}{C_{{\alpha_{m}}_{\lambda +\text{1}}}}(C_{{\alpha_{m}}_{\lambda +\text{1}}}-p_{{{\alpha_{m}}_{\lambda }}_{t}})\right)+\sum \left(p_{n_{{m_{\lambda }}_{t}}}\frac{{r_{n}}_{m_{\lambda }}}{C_{{\alpha_{m}}_{\lambda +\text{1}}}}(C_{{\alpha_{m}}_{\lambda +\text{1}}}-p_{{{n_{m}}_{\lambda }}_{t}})\right)+\sum p_{{\gamma_{m}}_{\lambda_{t+\text{1}}}}=\text{1} \end{array}$$ (3)

where $p_{{{\alpha_{m}}_{\lambda }}_{t}}$ and $p_{{{\alpha_{m}}_{\lambda }}_{t+\text{1}}}$ are the proportions of artifacts referred to the primary unit α at the time t and t+1 respectively, and $p_{n_{{m_{\lambda }}_{t}}}$ is the proportion of artifacts referred to the secondary unit *n*. $C_{{\alpha_{m}}_{\lambda +\text{1}}}$ is the maximal proportion of the primary unit in a set *m* at the taxonomic level λ+1, and ${r_{\alpha }}_{m_{\lambda }}$ and ${r_{n}}_{m_{\lambda }}$ are the replication rates of artifacts belonging to the primary unit  α and the secondary units *n*, respectively.

Parameter $\sum p_{{\gamma_{m}}_{\lambda_{t+\text{1}}}}$ is the sum of the proportions of innovations γ introduced to a cultural set. This parameter, capturing either slow cumulative changes or rapid transformations of material culture, requires additional explanation. In case of slow cumulative changes in an assemblage, the slight decrease of the primary unit’s proportion and unchanged proportions of the secondary units (one of which must eventually become primary according to the model assumptions) causes the introduction of one or several units represented in low proportions. Alternatively, rapid transformation of an assemblage corresponds to an increase of the primary unit’s proportion followed by its rapid drop. This decline in primary and secondary units is ‘compensated’ by the introduction of an innovation package, i.e., $\sum p_{{\gamma_{m}}_{\lambda_{t+\text{1}}}}>\text{0}$ [69] (for the examples from various fields see [70–72]).

Simulations applying logistic equations to cultural assemblages frequently assume their equilibrium state by setting the maximal proportion of a trait to a constant, i.e., $C_{max}=\text{1}$ [14]. We instead consider changes in the maximal absolute numbers of a trait to capture production bias and take into account the impact of the non-equilibrium state on assemblage diversities [28,32,73–75]. This is made possible by introducing a parameter *k*, which represents the amount of change in the total number of a trait behind the threshold *C* in a time span from t to t+1. Given that values of *p* and *C* do not exceed 1 (Equation 3), the variable *k* also calibrates the proportions of units in accordance to the amount of change in the maximal proportion of a trait: $\frac{p_{\alpha_{t}}}{k}$, $\frac{p_{\beta_{t}}}{k}$, $\frac{p_{n_{t}}}{k}$. This calibration approach preserves the *p*/*C* ratios set in Equation 3.

Since the logistic equation includes the equilibrium point at p/k=C/2, while $C_{max}$ and $p_{max}$ are limited to 1, values of the k-parameter are less than or equal to two. The values of *k* which do not exceed 1 reflect a decline in total production (= total consumption). The equilibrium point of k=1 indicates stable total production (= total consumption). The values of *k* which exceed the equilibrium point, but do not exceed 2, reflect an increase in total production (= total consumption).

Thus, integrating the above-described modifications, we generalize the original model as expressed in Equation 4. This equation captures the quantitative and qualitative changes in the assemblage composition, accounting for both the so-called ratchet effect, which describes the accumulation of technological and behavioral modifications [76,77]), in temporal meso-scale series and short-time rapid innovation burst (see [78] and references therein).


$$\begin{array}{c} D=\text{exp}\left(\sum \left(\frac{\text{1}}{k}p_{{{\alpha_{m}}_{\lambda }}_{t}}\frac{{r_{\alpha }}_{m_{\lambda }}}{C_{{\alpha_{m}}_{\lambda +\text{1}}}}\left(C_{{\alpha_{m}}_{\lambda +\text{1}}}-\frac{p_{{{\alpha_{m}}_{\lambda }}_{t}}}{k}\right)\right)+\sum \left(\frac{\text{1}}{k}p_{n_{{m_{\lambda }}_{t}}}\frac{{r_{n}}_{m_{\lambda }}}{C_{{\alpha_{m}}_{\lambda +\text{1}}}}\left(C_{{\alpha_{m}}_{\lambda +\text{1}}}-\frac{p_{{{n_{m}}_{\lambda }}_{t}}}{k}\right)\right)+\sum p_{{\gamma_{m}}_{\lambda_{t+\text{1}}}}\right) \end{array}$$

$$\begin{array}{c} \left(\sum p_{i}=\sum p_{\alpha }+\sum p_{n}+\sum p_{\gamma }=\text{1};\;p\leq k;\;k\leq \text{2};\;\text{0}\leq r\leq \text{4}\right) \end{array}$$ (4)

The proportion of the secondary units is controlled by the primary unit’s replication rate as follows:


$$\begin{array}{c} \sum {r_{n}}_{m_{\lambda }}\frac{p_{n_{{m_{\lambda }}_{t}}}\left(C_{{\alpha_{m}}_{\lambda +\text{1}}}-p_{n_{{m_{\lambda }}_{t}}}/k\right)}{p_{{{\alpha_{m}}_{\lambda }}_{t}}\left(C_{{\alpha_{m}}_{\lambda +\text{1}}}-p_{{{\alpha_{m}}_{\lambda }}_{t}}/k\right)}=\frac{kC_{{\alpha_{m}}_{\lambda +\text{1}}}}{p_{{{\alpha_{m}}_{\lambda }}_{t}}\left(C_{{\alpha_{m}}_{\lambda +\text{1}}}-p_{{{\alpha_{m}}_{\lambda }}_{t}}/k\right)}-{r_{\alpha }}_{m_{\lambda }} \end{array}$$ (5)

The stability-causing threshold indicates the cumulative effect of production and transmission of a particular trait, which does not change its proportion relative to the change in total production. This stability-causing threshold, i.e., the replication rate leading to an unchanged proportion of a primary unit α, is estimated as follows:


$$\begin{array}{c} r_{\alpha_{stab}}=\frac{kC_{{\alpha_{m}}_{\lambda +\text{1}}}}{\left(C_{{\alpha_{m}}_{\lambda +\text{1}}}-p_{{{\alpha_{m}}_{\lambda }}_{t}}/k\right)} \end{array}$$ (6)

Any trait may gain or abandon its selective value over time. Unbiased transmission presumes that $p_{{{\alpha_{m}}_{\lambda }}_{t}}\approx p_{{{\alpha_{m}}_{\lambda }}_{t+\text{1}}}$ [49]. Therefore, according to Equation 4, replication rate ${r_{\alpha }}_{m_{\lambda }}$, which is equal to or approaching $r_{\alpha_{stab}}$, is indicative for a style, whereas replication rate ${r_{\alpha }}_{m_{\lambda }}$, which is sufficiently lower or higher than $r_{\alpha_{stab}}$, is indicative for a function (frequency-dependent bias). Note that, according to Equation 5, the proportion and replication rate of the secondary units, ${r_{n}}_{m_{\lambda }}$ are also conditioned by the values of ${r_{\alpha }}_{m_{\lambda }}$. Replication rate ${r_{\alpha }}_{m_{\lambda }}$ exceeding $r_{\alpha_{stab}}$ obviously leads to the increase of the proportion of a taxonomical unit. Values of ${r_{\alpha }}_{m_{\lambda }}$ below $r_{\alpha_{stab}}$ cause the decrease of the proportion of a unit, which result in a shift of the unit’s category from the primary to the secondary.

Transforming Equation 6 allows estimation of the other threshold, the maximal proportion of the primary unit:


$$\begin{array}{c} p_{{{\alpha_{m}}_{\lambda }}_{t}}\leq kC_{{\alpha_{m}}_{\lambda +\text{1}}}-\frac{k^{\text{2}}C_{{\alpha_{m}}_{\lambda +\text{1}}}}{r_{\alpha_{stab}}} \end{array}$$ (7)

The following section investigates the interplay between the model parameters and their impact on the assemblage diversities.

## Simulations and results

A rough scheme categorizing material sets according to their internal variation includes four units. Low and high proportions of traits do not significantly impact assemblage diversity [17]. The highest contribution to a diversity measure is provided by the units with proportions ranging between c. 0.25 and 0.5 (Fig 2). According to the model assumptions, the proportion of the primary unit exceeds the proportion of any secondary units before the former starts dropping. Since diversity increases with the increasing number of units [68], a lower proportion of the primary unit presumes a higher number of the secondary units and higher diversity. Therefore, assemblages with the proportion of the primary unit below and above $C_{{\alpha_{m}}_{\lambda +\text{1}}}/\text{2}$ are labeled as, respectively, ‘rather more diverse’ (RMD) and ‘rather less diverse’ (RLD). Assemblages with the proportion of the primary unit below 0.2 and above 0.8 are labeled as, respectively, ‘more diverse’ (MD) and ‘less diverse’ (LD). We caution the reader that the assemblage diversity is impacted by the number and proportions of all traits, including the secondary ones, and, therefore, our conditional labeling does not perfectly match the actual diversity of material culture sets.

**Fig 2:**
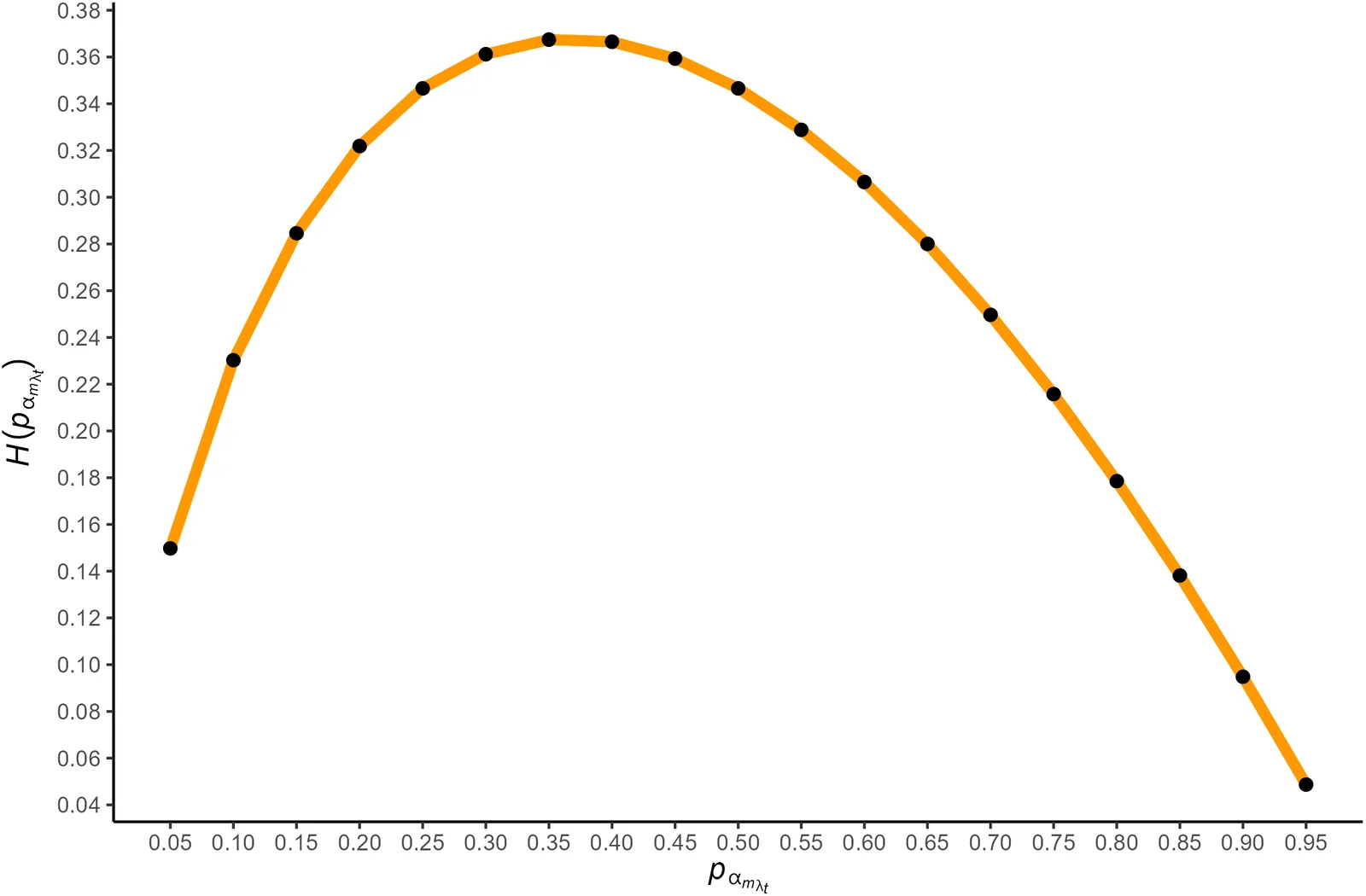
Contribution of the primary unit to the entropy of the assemblage.

### Total production and the maximal proportion of a trait

Equation 7 identifies the maximal proportion of the primary unit maintained by stability and change in the total production. The maximal unit proportion increases from bottom to top of the taxonomic hierarchy with the increase in $C_{{\alpha_{m}}_{\lambda +\text{1}}}$ and decrease in $\frac{{r_{\alpha }}_{m_{\lambda }}}{C_{{\alpha_{m}}_{\lambda +\text{1}}}}$ in the range limited by $C_{{\alpha_{m}}_{\lambda +\text{1}}}=\text{1}$ and ${r_{\alpha }}_{m_{\lambda }}=r_{stab}=\text{4}$ (Fig 3). The linear increase of the k-parameter representing the slowing decline (k<1), stability (k=1), or increase (1<k≤2) in the total production corresponds to the exponential increase of the maximum proportion of the primary unit. Stability in the total production limits the maximum proportion of the primary unit to 0.75. The maintenance of the primary unit’s stable proportion exceeding this value over a substantial time is made possible only by the increase in total production. The highest proportion of the primary unit, corresponding to zero assemblage diversity ($p_{{{\alpha_{m}}_{\lambda }}_{t}}=\text{1}$), is maintained by the highest possible increase in total production (k=2).

**Fig 3:**
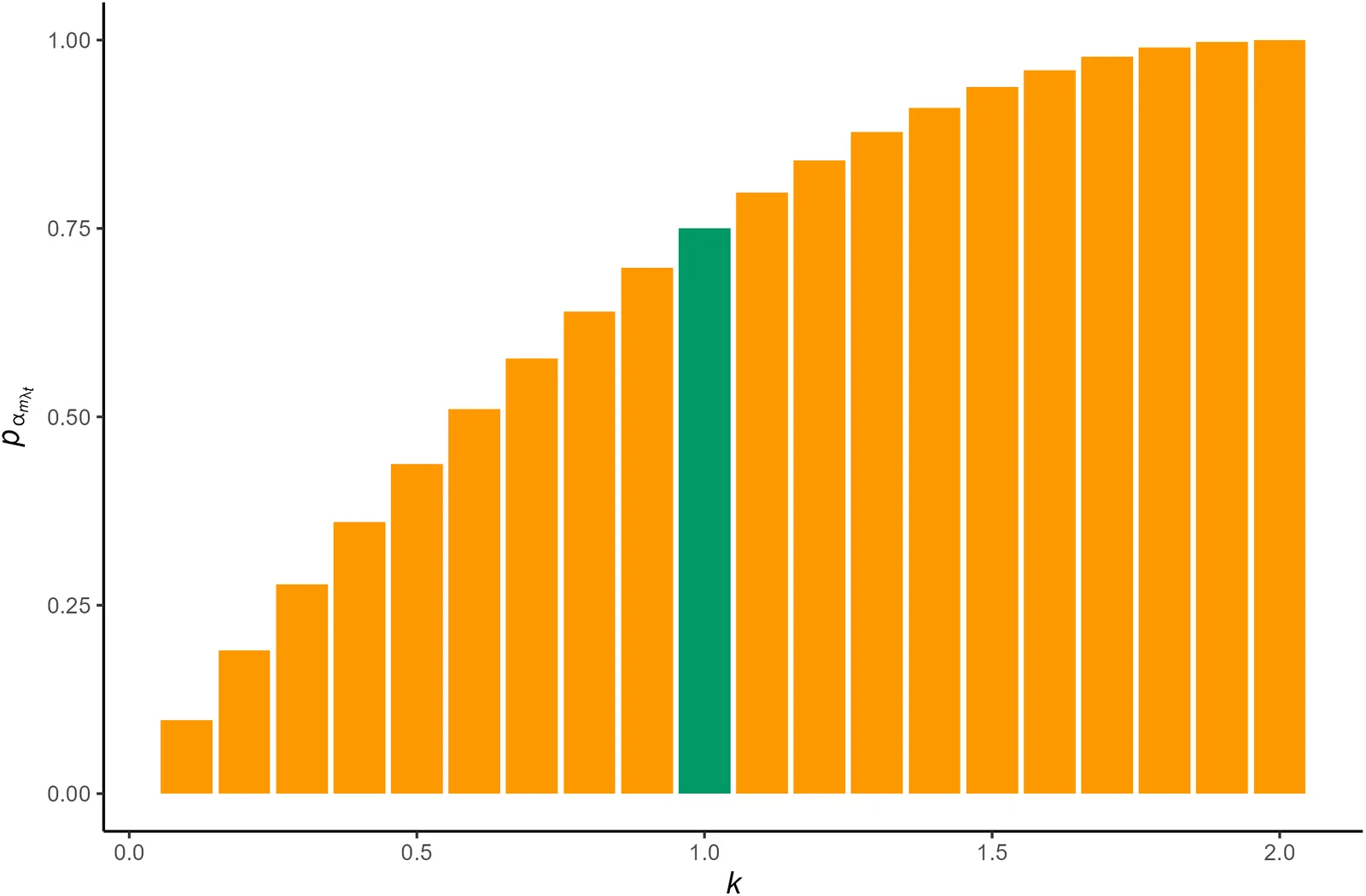
Maximal stable proportion of the primary unit maintained by production stability (k=1) and change (k≠1).

In case the total production does not further increase, the proportion of the primary unit may exceed thresholds represented in Fig 3 only for a single unit of time, t. At the subsequent time unit (t + 1), the primary unit turns into the secondary unit due to its decreasing proportion (Equation 4). Accordingly, assemblage diversity decreases in time t and then increases in (t + 1). For example, consider a hypothetical assemblage composed of types A, B, and C distributed at proportion of, respectively, 0.8, 0.1 and 0.1 (Fig 4). The increase in the total production is described by k=1.1, so that the proportion of the primary unit slightly exceeds the threshold 0.7975 corresponding to the value of k. The replication rate of the primary unit is set to 3.6, while the replication rate of the secondary units B and C (assumed to be equal) is estimated with the Equation 5 is ${r_{n}}_{m_{\lambda }}=\text{1}.\text{7}\text{3}$. The proportion of A decreases to c. 0.714 and the proportion of B and C increases to c. 0.143 from t to (t + 1). Accordingly, the Shannon diversity (the number of ‘effective’ types) of the hypothetical assemblage increases from c. 1.81 to c. 2.22 (Fig 4).

**Fig 4:**
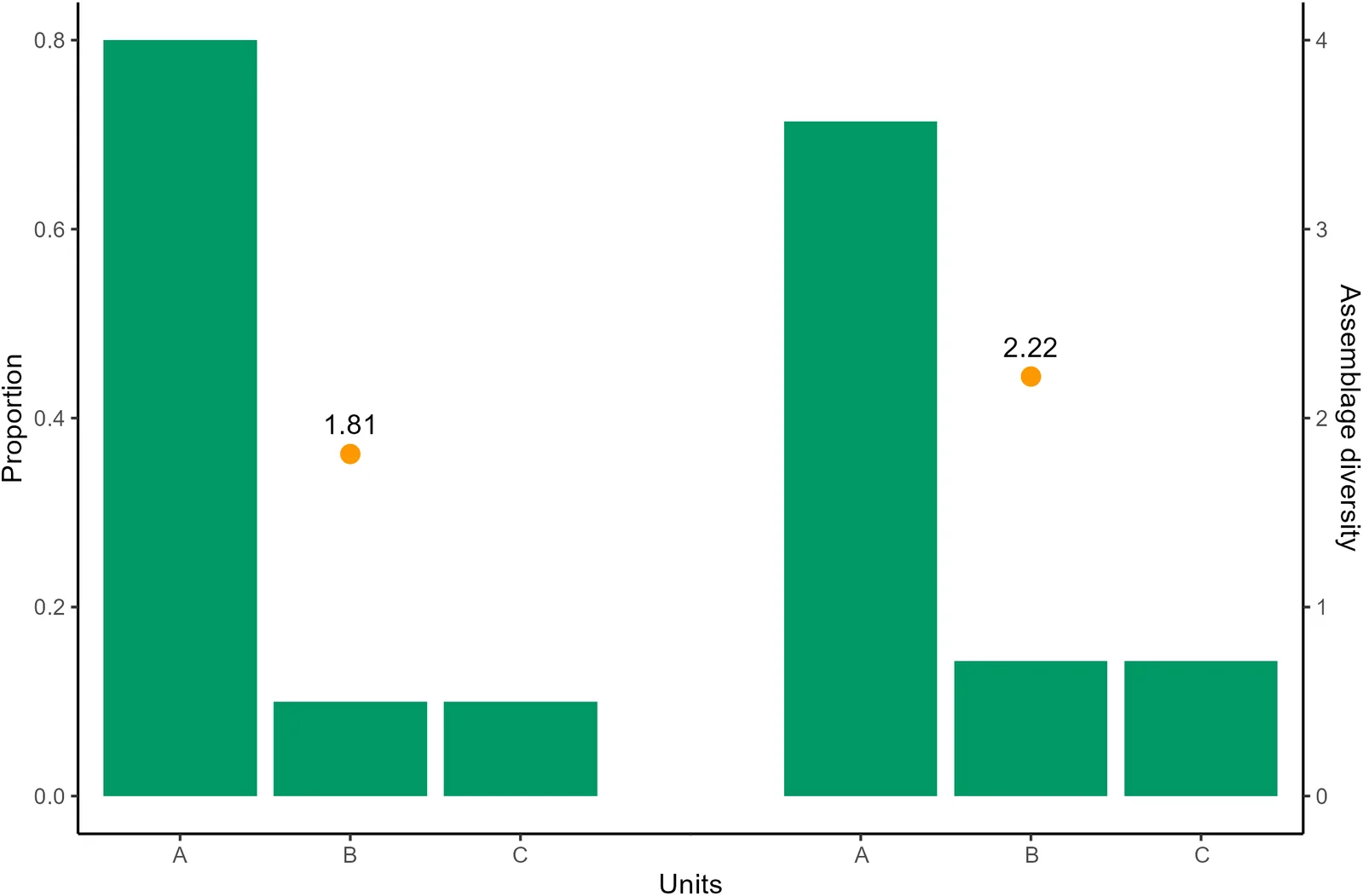
Hypothetical change in the proportion of artifact styles estimated with Equation 4 (histograms) and associated change in the assemblage diversity (dots).

### Assemblage diversity and increasing production

The relationship between the replication rates required for maintaining a stable proportion of the primary unit and increasing total production exhibits path-dependency in assemblage diversity. The linear increase of the k-parameter corresponds to the near-linear increase of the replication rate supporting the stable proportion of the primary unit in RMD assemblages, which is exemplified by the value of $p_{{{\alpha_{m}}_{\lambda }}_{t}}$ set to 0.25 (Fig 5a). In RLD assemblages, exemplified by the value of $p_{{{\alpha_{m}}_{\lambda }}_{t}}$ set to 0.75, the increasing total production is associated with decrease in the required replication rate of traits up to nearly the middle of the k-parameter’s range. As the values of the k-parameter further increase, replication rates required for maintaining a stable proportion of the primary trait also increase (Fig 5a).

**Fig 5:**
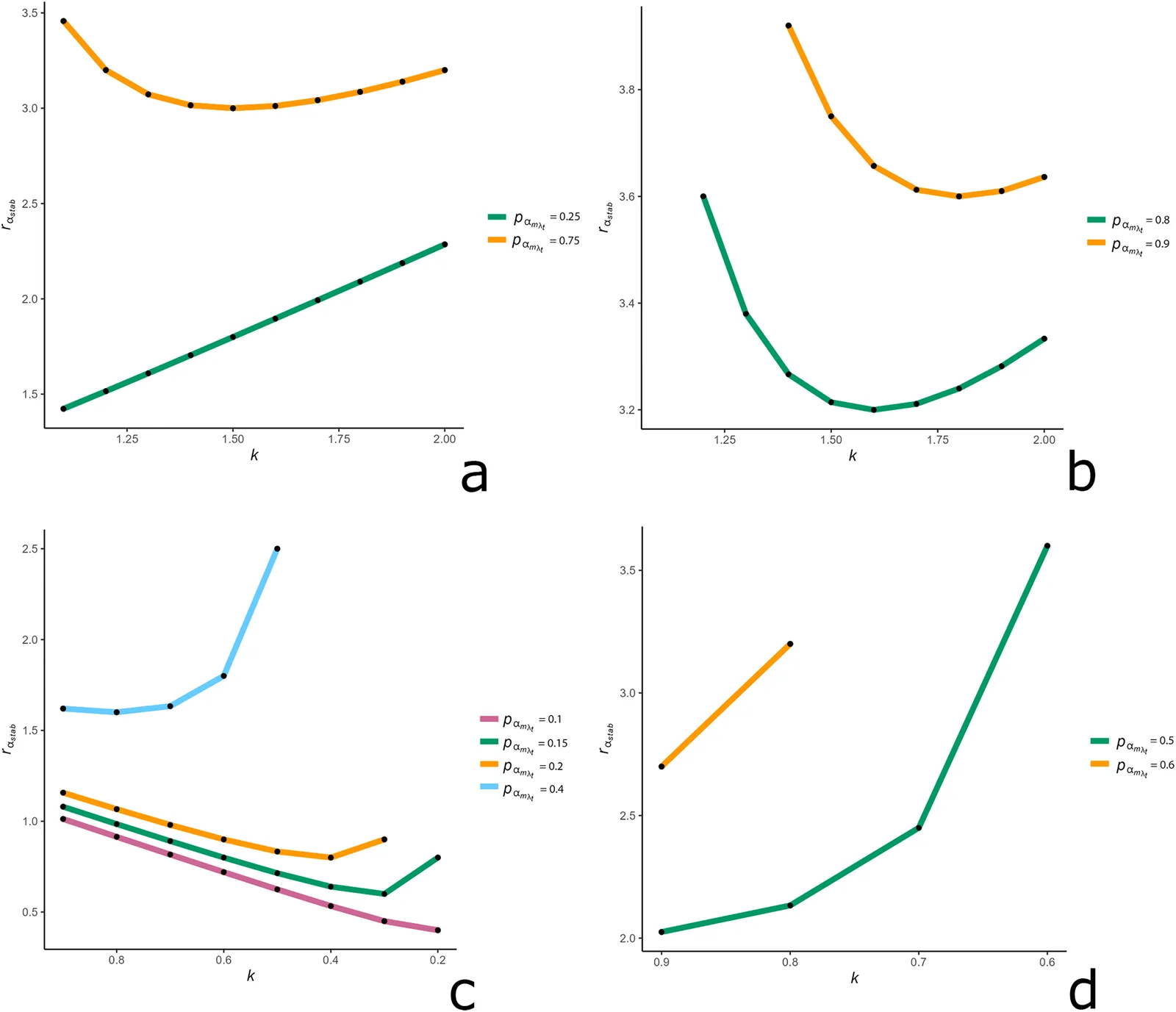
Replication rate supporting the stable proportions of the primary unit in archaeological assemblages under increasing or decreasing total production. a – Replication rate supporting the stable proportions of the primary unit ($r_{\alpha_{stab}}$) in rather more diverse and rather less diverse sets under increasing total production (1<k≤2); b – replication rate supporting the stable proportions of the primary unit ($r_{\alpha_{stab}}$) in more diverse sets under increasing total production (1<k≤2); c, d – replication rate supporting the stable proportions of the primary unit ($r_{\alpha_{stab}}$) with decreasing total production (k<1) in more diverse and rather more diverse sets (c), and rather less diverse sets (d).

Fig 5b exemplifies MD sets by the stable proportion of the primary unit set to 0.8 and 0.9. It is notable that the small increase in the total production is associated with the values of $r_{\alpha_{stab}}$ exceeding the maximal possible replication rate. More specifically, $r_{\alpha_{stab}}=\text{4}.\text{03 }$ is associated with k=1.1 for $p_{{{\alpha_{m}}_{\lambda }}_{t}}=\text{0}.\text{8}$, and $r_{\alpha_{stab}}$ equal to 6.05, 4.8 and, c. 4.23 is associated with values of k set to 1.1, 1.2, and 1.3, respectively, for $p_{{{\alpha_{m}}_{\lambda }}_{t}}=\text{0}.\text{9}$. Since values of $r_{\alpha_{stab}}$ exceeding 4 are not allowed by the model, the proportions of the primary unit in the MD sets are necessarily decreasing, while the assemblage diversity is necessarily increasing with a relatively small increase in total production. A more intensive increase in total production enables maintenance of the proportion of traits in the MD sets under relatively high replication rates (Fig 5b).

### Assemblage diversity and declining production

Path-dependency is also notable for the decline of total production. The possibility of maintaining stable proportions of the primary unit increases with decreasing proportions of this unit (Fig 5c and 5d). The linear decline of the k-parameter requires the nearly linear decline of the $r_{\alpha_{stab}}$ until its values reach a certain critical point. After reaching this critical point, the MD diverse sets with the lowest proportion of the primary unit (for example, $p_{{{\alpha_{m}}_{\lambda }}_{t}}=\text{0}.\text{1}$), reveal slowing decline in the values of $r_{\alpha_{stab}}$ towards the most rapid decline in total production, i.e., from k=0.3 to k=0.2 (Fig 5c). In the MD assemblages with higher proportion of the primary unit (for example, the proportions of $p_{{{\alpha_{m}}_{\lambda }}_{t}}$ equal to 0.15 and 0.2), as well as the RMD and RLD sets (for example, the proportions of $p_{{{\alpha_{m}}_{\lambda }}_{t}}$ equal to 0.4, 0.5, and 0.6), values of $r_{\alpha_{stab}}$ demonstrate a shift from decline to exponential increase after reaching a critical point (Fig 5c and 5d).

Replication rates of the primary unit which are either smaller or higher than the values of $r_{\alpha_{stab}}$ represented in Fig 5 cause respective decrease in the proportions of the primary unit, or their increase followed by the decrease (see Fig 4). Assemblage diversity increases with the decreasing proportion of the primary unit, and decreases with its increase. A stable proportion of the primary unit in the LD sets in the case of decreasing total production is generally not allowed by the model.

### Cycles

The interrelationship between the model parameters describes various possible scenarios for short-term fluctuations and cycles in the assemblage diversity, which are caused by an individual or a mutual impact of production and cultural transmission biases. Changes in the total production impact the values of two thresholds framing the model behavior. The first threshold is $r_{\alpha_{stab}}$. RMD and MD sets tend to decrease in diversity. A minimal and maximal increase or decline in total production causes a significant growth or decline of $r_{\alpha_{stab}}$; an ‘average’ increase or decline in production causes a moderate growth or decline of $r_{\alpha_{stab}}$ in RLD and LD sets. Replication rates below this threshold force such assemblages to increase in diversity. Also, increasing or declining total production sets the threshold of the maximal proportion of a trait, $p_{{{\alpha_{m}}_{\lambda }}_{max}}$. As the proportion of the primary unit exceeds this threshold, assemblage diversity must decline and then increase. Both thresholds frame rise-and-fall patterns in assemblage diversity, either when the total production is being changed, or remains stable. The model behavior reveals a notable trend of correlation between the increasing total production and stable or increasing highest proportion of primary units ($p_{{{\alpha_{m}}_{\lambda }}_{max}}$ increases at a higher rate than $p_{{{\alpha_{m}}_{\lambda }}_{t}}$), i.e., standardization of traits resulting from increasing total production.

A few of the numerous scenarios linking assemblage diversity to population boom-and-busts and production biases are illustrated as follows. Assume that the total production is linearly proportional to the hypothetical population proxy, so that the number of producers increases or decreases at a slower rate than the number of consumers (Fig 6 and Table 1). Both population and production remain stable at the beginning of a cycle (time units 1 and 2) and then exponentially increase until the time unit 5. Slowing population growth at time unit 6 is followed by population decline in time units 7 and 8. The very beginning of a cycle is characterized by the unified material culture set including 0.8 traits of the hypothetically ‘older’ type A and traits of the ‘novel’ types B, C, and D distributed at 0.1, 0.05, and 0.05, respectively. The replication rate of the secondary types (right side of Equation 5) is distributed proportionally to their frequencies in the population in time units 1 and 2 (Fig 6).

**Fig 6:**
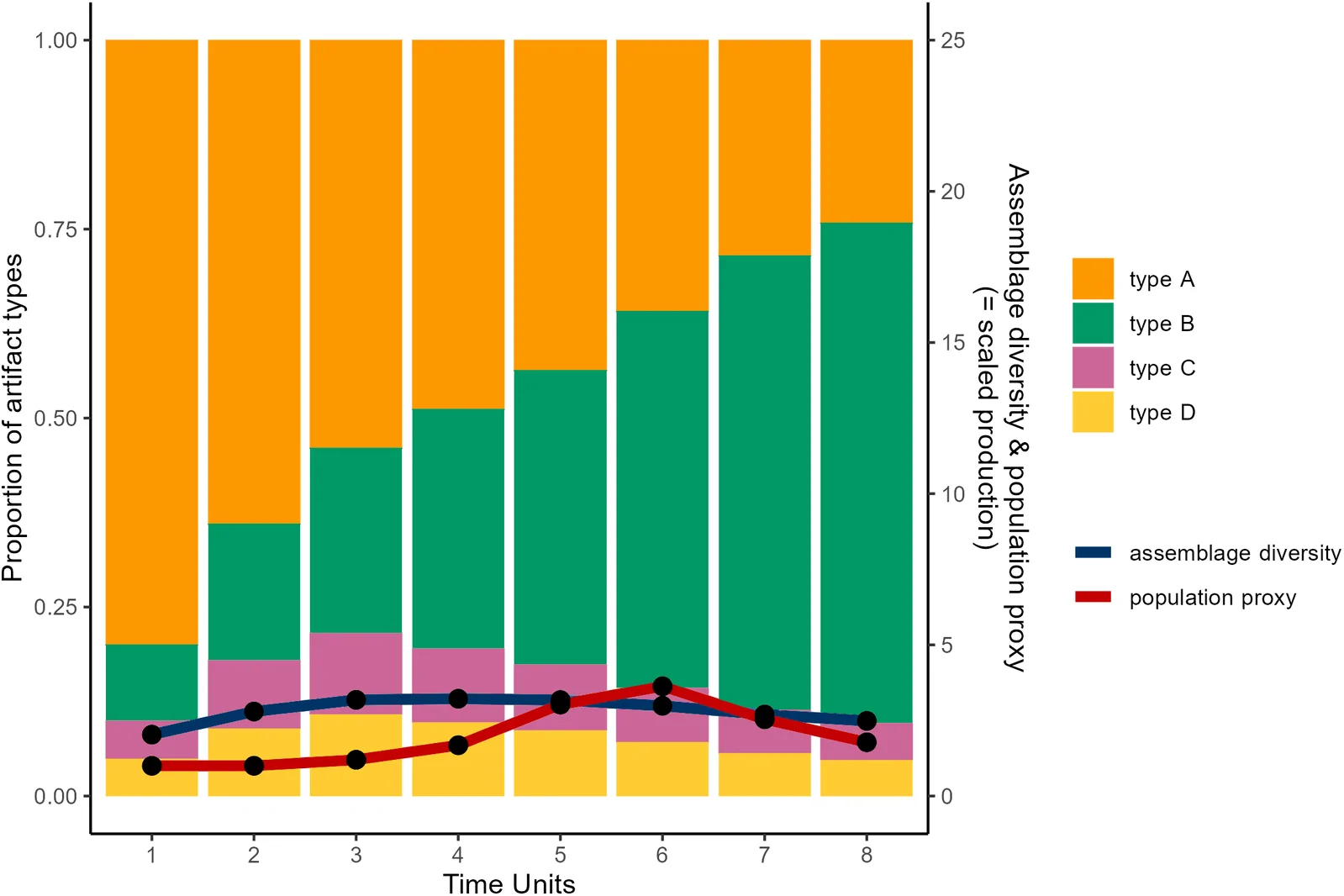
Hypothetical population, production, and assemblage diversity cycles under moderate pro-novelty bias. Total production is proportional to the population size. The histogram represents the proportion of cultural traits in a set.

Further simulation inputs are based on the theoretical hypothesis that in the course of a sociopolitical cycle, the aggregate concept of social diversity or meta-identity undergoes a wear-off effect as the cycle shifts from the integrative to the disintegrative phase [4]. In the comparatively well-researched LBK context, this changing meta-identity would be marked by changes in band-type decoration techniques, but also in the disappearance of the typical figurines after the early phases (Fig 7) [25,80]. Accordingly, the model shows that the replication of traits is biased towards novel types. The ‘novel’ primary traits increase at the rate of $(r_{\alpha_{stab}}+\text{0}.\text{5}$) and the replication rate of the ‘older’ secondary type is lower than the replication rate of ‘novel’ secondary types comparing to the frequencies of these types in the set starting from the time unit 3. More specifically, proportions of the summarized replication rates in the right side of Equation 5 are unevenly distributed among the ‘older’ and ‘novel’ units.

**Fig 7:**
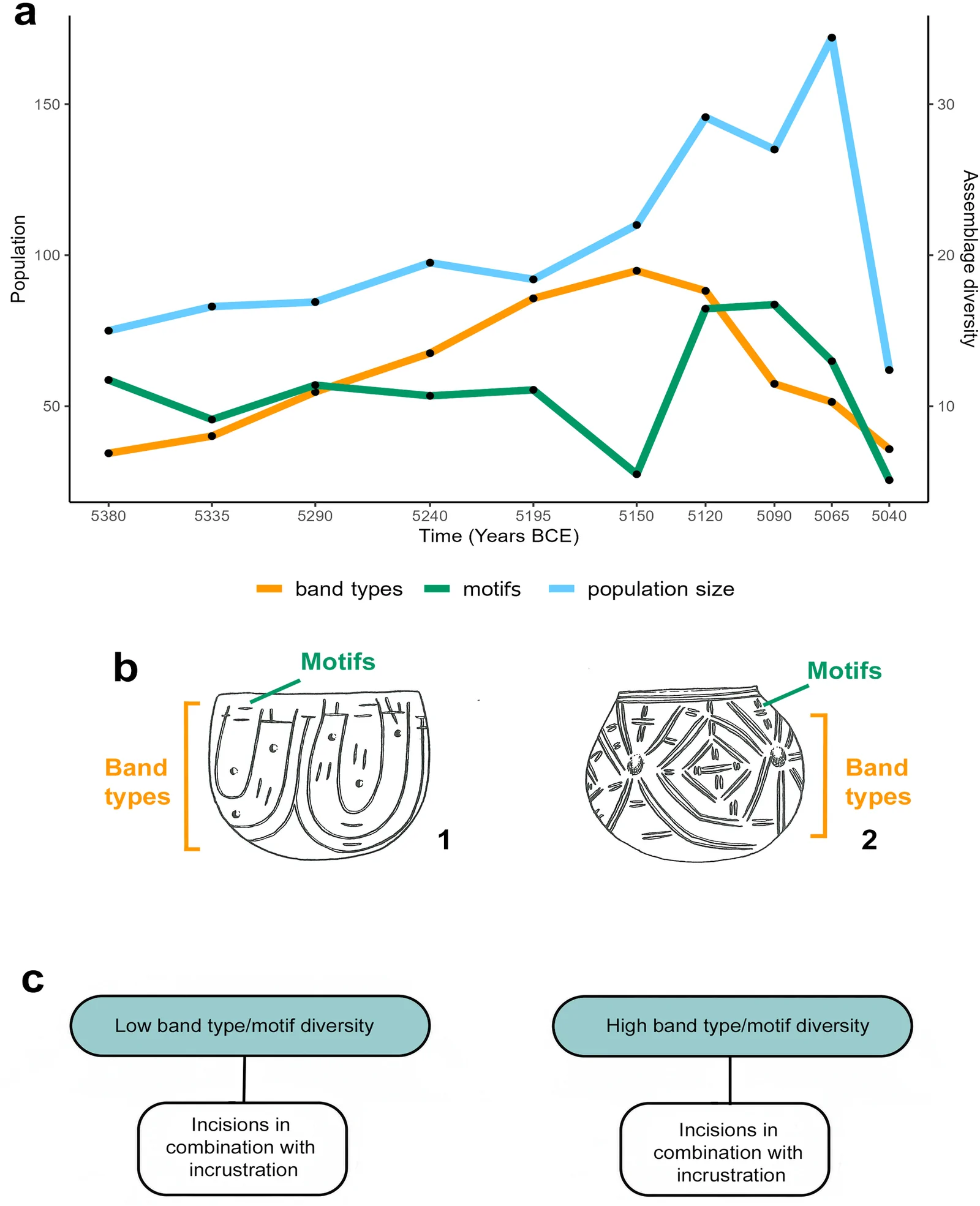
Linear Pottery Culture (LBK) in south-western Central Europe: Population proxy and diversity estimates of the primary and secondary decoration motifs, 5400 – 5000 BCE. a – graph based on data from [4,25]; b – 1 – early LBK vessel, 2 – late LBK vessel (both from [79]); c – decoration modes.

Changes in the assemblage diversity in time unit 2 are exclusively caused by the cultural transmission biases due to stable production. Further dynamics result from the feedback effect between the production and transmission biases. Diversity of the set increases until time unit 4 and starts decreasing at time unit 5, before the population reaches its peak. Assemblage diversity continues decreasing with declining population during time units 7 and 8.

Given that all other parameters remain unchanged, the accelerated transmission biases result in intensified changes of assemblage diversity. This is illustrated by the increasing replication rate of type B in time unit 2, disproportional to its frequency compared to the frequencies of C and D, and the increasing replication rate of the primary unit in our hypothetical set to ($r_{\alpha_{stab}}+\text{0}.\text{7})$ starting from the time unit 3 (Fig 8). Assemblage diversity shifts towards decrease one time unit earlier (cf. Figs 6 and 8). Declining population and total production in the time unit 7 causes singularity in model equations, so that any value of ${r_{\alpha }}_{m_{\lambda }}$ exceeding zero result in the negative values of $p_{{{\alpha_{m}}_{\lambda }}_{t}}$. Due to the operation of mechanisms which are not covered by the model equations (i.e., are correspondent to the singularity), we assume further decrease in the secondary type A and extinction of the types C and D, introducing the novel types E (become primary type), F, G, H, I, and J to the set (Fig 8: time unit 7). This replacement of types corresponds to the increase in assemblage diversity.

**Fig 8:**
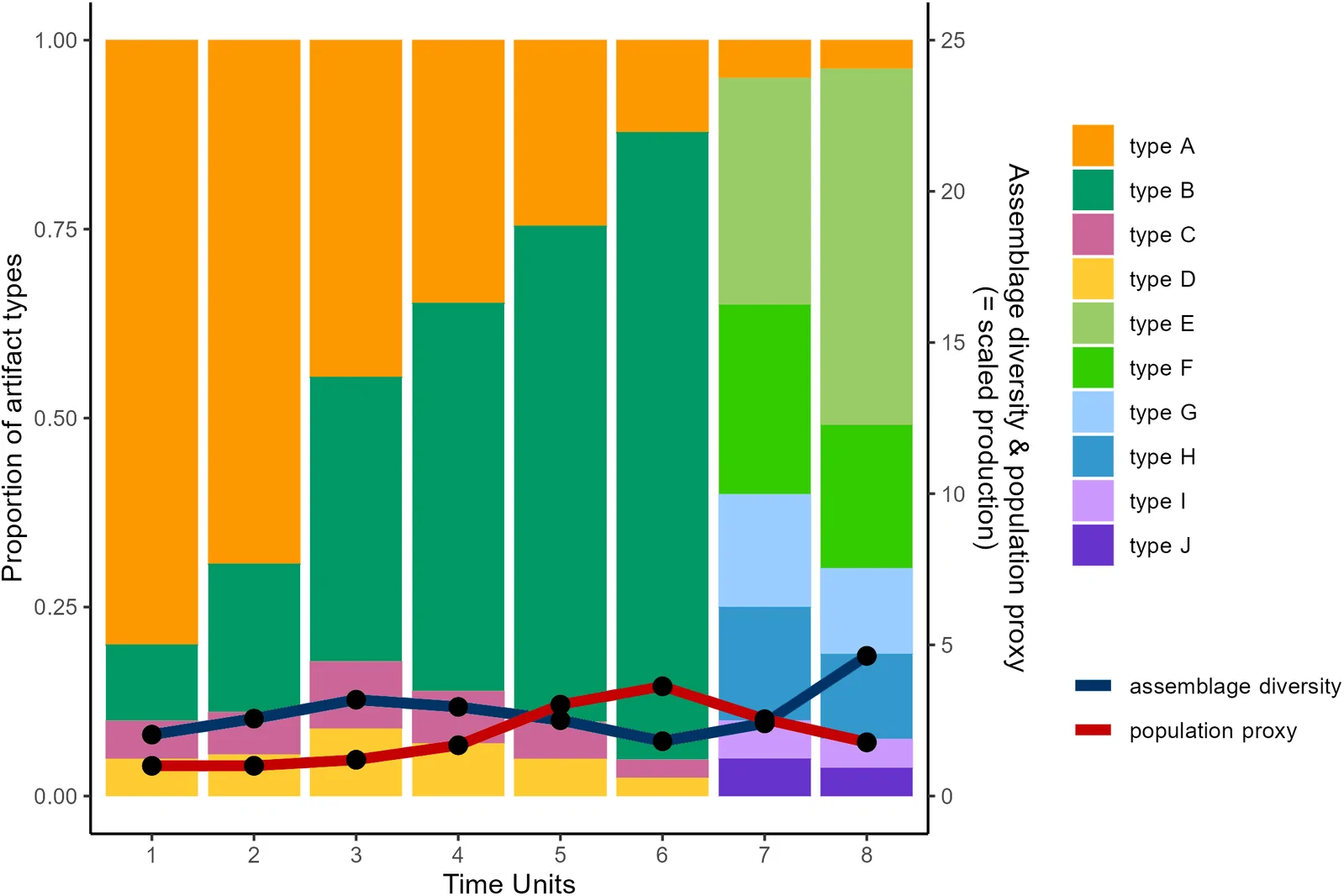
Hypothetical population, production, and assemblage diversity cycles under accelerated increase in the primary type and moderate pro-novelty bias in the secondary types. Total production is proportional to the population size. The histogram represents the proportion of cultural traits in a set.

Next, given that all other initial parameters remain unchanged (including the accelerated growth of type B in the time unit 2), we replace the population boom-and-bust with an exponential increase. Fig 9 exemplifies the extreme case of a single type remaining in the set by the end of the cycle. The dynamics in the time units 1–5 represented in Fig 8 repeat the pattern shown in Fig 8. However, a shift of the total production to an accelerated increase in time units 6–8 corresponds to decreasing assemblage diversity (i.e., standardization).

**Fig 9:**
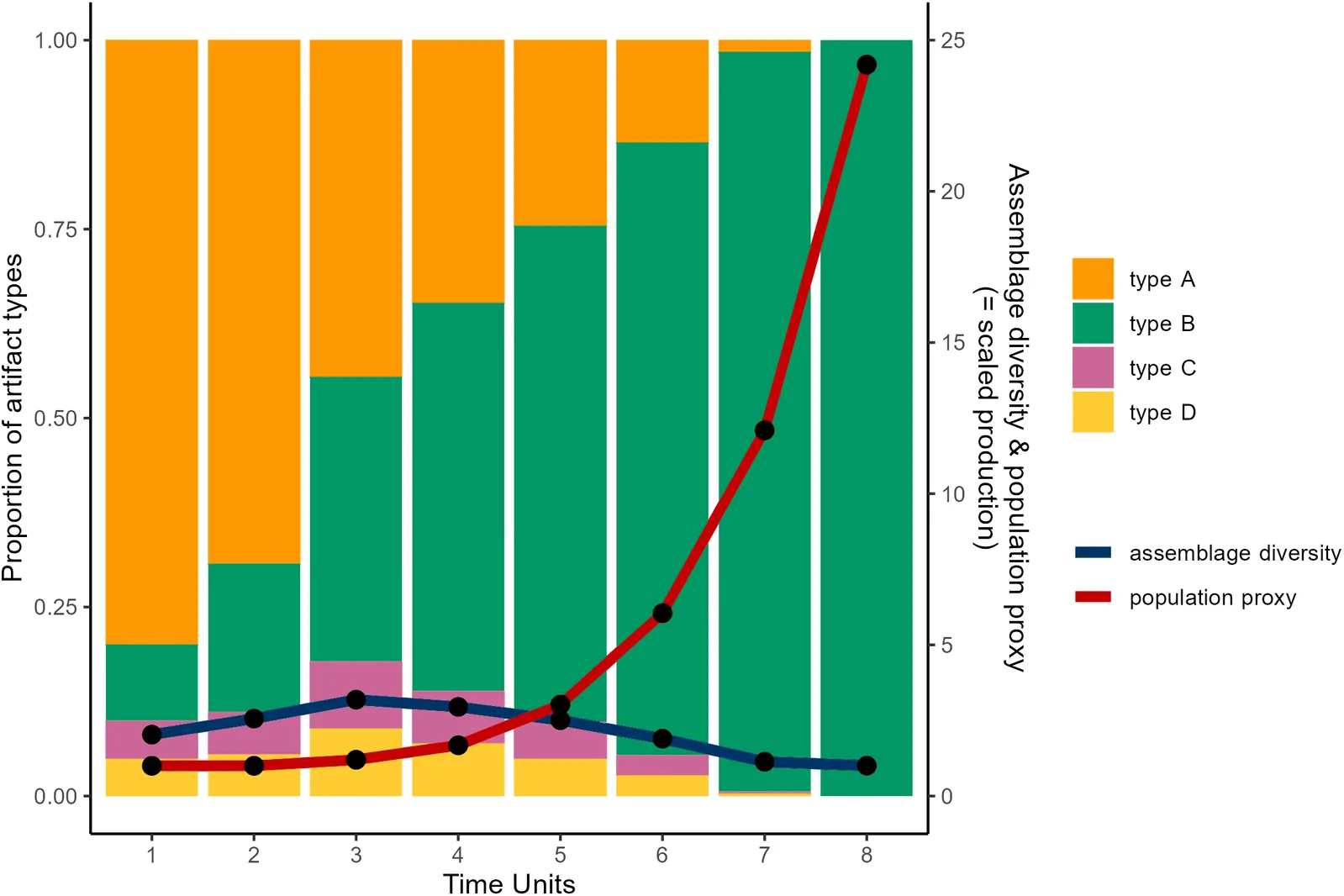
Hypothetical assemblage diversity cycle resulting from increasing population and production. Total production is proportional to the population size. The histogram represents the proportion of cultural traits in a set.

## Discussion

The outcome of our model demonstrates a need for a shift from pure biological metaphors often underlying hypotheses of neutrality and transmission biases towards social and technological aspects of production and consumption. Pioneering microevolutionary approaches ‘translating’ a genetic methodology into the language of archaeology significantly advanced our knowledge on culture change; this knowledge is being extended and improved by contextualizing cultural transmission with production biases and changing social environments [81–85].

Equilibrium production results from the unchanged proportion of producers and consumers. While this equilibrium (production stability) holds, the innovation and cultural transmission biases are the only factors of material culture change (Table 1). A replication rate *in all traits* approximately equal to the stability-causing threshold $r_{\alpha_{stab}}$ corresponds to an unbiased cultural transmission describing the replication of traits proportionally to their frequency in a population [14,49]. In this case, assemblage diversity may gradually change due to innovation (cultural mutation), stochastic processes, and the structure of social networks [14,47,86–88]. Replication rates which are disproportional to the frequency of a trait in an assemblage and, therefore, shifted from $r_{\alpha_{stab}}$ (transmission biases), result in a more intensive change of the assemblage diversity.

A mutual impact of the non-equilibrium production and transmission biases on the variation in material culture sets is probably the most common case in analyses of meso- and long-term patterns. This may be illustrated by the similarity of the model behavior captured in Fig 6 and the diversity cycle of the LBK primary decoration motifs in Neolithic south-western Central Europe (Fig 7). Our model explains the increasing assemblage diversity by population increase, social complexity increase reflected in accelerated production, and a pro-novelty transmission bias. Due to these factors, cumulative changes in the diversity of the primary decoration motifs reach their terminal stage during a shift to decreasing diversity (i.e., the decreasing manifestation of meta-identity) prior to the population decline (Fig 7). Therefore, the corresponding further increase of the diversity of the secondary decoration motifs indicates a gradual re-focus from the manifestation of a meta-identity to the manifestation of the self-identification of social sub-groups.

Alternatively to models describing a relatively gradual change in the assemblage composition and culture history approaches associating rapid replacement of trait packages with migrations, our model allows for the description of the simultaneous replacement of [almost] all trait types in the same population due to the specific interplay of the production and cultural transmission biases (Fig 8). According to the model, stable or increasing production could cause a drop in the proportion of the primary unit. The replacement of assemblage components occurs as the result of an earlier intensified production under accelerated pro-novelty bias, followed by declining production. An empirical example of the [almost] complete replacement of traits with the changing of archaeological cultures is the replacement of the latest Cucuteni-Trypillia units by the Yamnaya (also known as Yamna) and Globular Amphorae culture in present-day Republic of Moldova and Ukraine after 3100/2950 BCE (hereinafter the chronology of Cucuteni-Trypillia and its component parts follows [89,90]). Both subsequent cultural units lack Cucuteni-Trypillia ‘ancestry’ in their material assemblages [91], with a possible exception of the Yamnaya stellae origin in the Usatovo group [92].

The chaotic properties (sensitive dependence upon initial conditions) of our model are worth mentioning. The complete disappearance of the primary trait represented in Fig 8 is partially affected by the transmission bias in time unit 2 compared to the simulation represented in Fig 6. More specifically, the replication rate of the ‘novel type’ B in the right side of Equation 5 increased from 0.5 (proportional to the frequency of the secondary types in a set) to 5/7 (disproportionally exceeding the frequencies of secondary types). If the proportional increase in the replication of secondary types in time unit 2 stays the same, our hypothetical set could maintain a proportion of up to 0.197 type B traits in the time unit 7. Thus, the delayed effects of the short-term fluctuations and stochastic processes in the meso- and long-term patterns must be kept in mind.

Our simulations found standardization (persistence of the primary trait in an increasing proportion over a substantial period of time) to be an outcome of increasing total production. Whether transmission biases are involved or not, accelerated production exceeding the replication rate of traits results in decreasing assemblage diversity. Being empirically confirmed (see below), this component of the model behavior contradicts an expectation of increasing artifact richness with increasing sample size [14,31,93,94]. In this respect, the model outcomes reflect the results of simulating the object-mediated cultural transmission (i.e., cultural transmission via public manifestation of cultural traits) by Crema et al. [81]. Their simulations did not find the richness of traits expected from the Wright-Fisher model application to cultural data under the condition of an increasing replication rate (original models: [95,96]; review of the applications to culture change: [51]). In other words, an identical increase in the proportion of a trait may be caused by intensified total production (Table 1) or by conformist transmission bias shifting producers to replicate a certain trait disproportionally to its frequency in population (for the conformist transmission, see: [28,49,97–99]).

While being quite intensively explored in archaeology [39–43,100–107], standardization as a production bias is often overlooked by models of culture change that may lead to a possible misinterpretation of the observed trends [81]. At the broadest scale, standardization – occurring as an increase in uniformity with increasing replication rates – is associated with a change in the organization of production coming with economic specialization and, therefore, consumer-producer ratios (e.g., [41] and references therein). Scaling to the individual unit of production (e.g., individual craftsman or workshop), the increase in replication rates results in a decreasing amount of time invested in a trait’s production. This ‘time-saving’ leads to a fall in the variation of traits produced by an individual unit below the inter-unit variability, which is confirmed by the empirical evidence [105–107]. However, we should underline that the association of standardization and increasing social complexity requires a certain caution. Intentional factors causing standardization may result from a general increase in the production scale but may not be associated with a change in the consumer-producer ratio [100,102].

Turning back to the above-cited LBK example, the discussion of socio-cultural dynamics behind the cycles in the secondary decoration motif time-series (Fig 7) is complicated because of the simplification we allowed for setting the beta diversity to 1 (see above). Since the secondary decoration motifs might be correlated with clusters of houses, alpha diversity for such groups approaches 1 and gamma diversity is nearly proportional to beta diversity. One potential explanation may be derived by extrapolating the presented model to the population subsets; the expectation would then be that increasing production within a house group results in the standardization of household production. Slowing production due to population decline causes a sudden drop or extinction of a decoration motif, which is potentially replaced by the most closely observed decoration motif, i.e., derived from the nearest neighbors [47]. The other possible explanation is a nesting effect, i.e., a rise-and-fall in diversity at the higher taxonomic level which tends to overlap with several cycles at the lower taxonomic level. Our approach preserves, among others, the properties of such nesting effects while generalizing the earlier model of self-organized cultural cycles in hierarchically organized traits. However, the estimation of alpha, beta, and gamma diversities and nesting effects resulting from the taxonomic structure of archaeological data are beyond the scope of this study. Therefore, we leave the aforementioned potential explanations as hypotheses for future research. One other possible explanation would link the cycles in the secondary decoration motifs in south-western Central Europe with the population decline following a growth peak. Associated production biases initiate rapid transformations of material culture sets, starting a new diversity cycle.

In contrast to the LBK example, beta diversity for the WTC assemblages in the Southern Bug and Dnieper interfluve approaches 1, and gamma diversity is nearly proportional to alpha diversity [10]. Geophysical plans of settlements from this region and other WTC areas reveal the approximately constant ratio of one pottery kiln per 20 dwellings at least between c. 3900 and 3650 BCE [108,109]. The overall increase in regional population (e.g., [38] based on [110,111]) would result in a gradual increase in production and a gradually increasing consumer-producer ratio. Unlike the pottery morphology, the number (exponentiated richness) of decoration classes may be caused by the production bias shifting the assemblages towards decreasing diversity [22] (Fig 10). According to Sergey Ryzhov, six classes are present for the assemblages dated to c. 4100–4000 BCE (incised decoration, monochrome and bichrome painting, combination of incisions with monochrome, bichrome and polychrome painting) [112,113]. By c. 3950 BCE, two of these classes (combination of incisions with bichrome and polychrome painting) went extinct. Furthermore, the decreasing diversity of decoration classes is caused by the disappearance of incisions combined with monochrome painting by c. 3900 BCE and the disappearance of incised decoration and bichrome painting by c. 3850 BCE [113–115].

**Fig 10:**
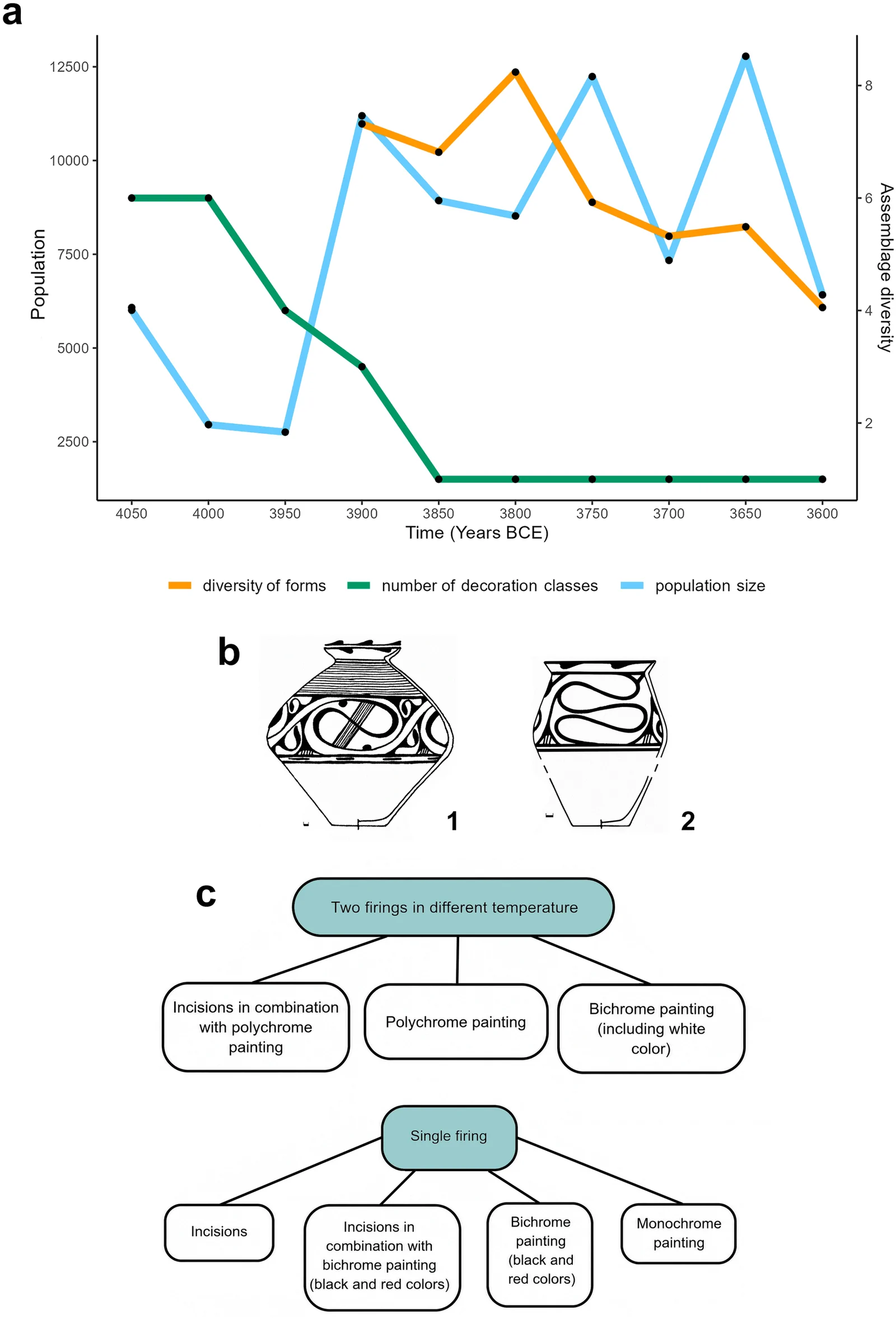
Assemblage diversity and population size in Western Trypillia groups in the Southern Buh and Dnipro interfluve, 4100 – 3600 BCE. a – population size (based on data from [38], diversity of pottery morphology (based on data from [10]), and richness (number) of decoration classes (based on data from [112–115]); b – example of vessel forms: sphero-conical vessels (1) were gradually replaced by biconical vessels (2): 1 and 2 – vessels from Kolodyste 2 site, adopted from [116]; c – number of vessels’ firings depending on the decoration class.

A trajectory from the variability in pottery ornamentation towards monochrome black painting through the decrease in the combination of incising and painting, and the number of colors demonstrates ‘time-saving’. Also, this trajectory is indicative of the decrease in the use of wood as fuel for vessel firing. The frequency of the most time-consuming combination of incisions and polychrome painting decreases between c. 4100 and 4000 BCE, while the proportion of incised decoration (not combined with painting) increases between c. 4000 and 3950 BCE [112,113]. These changes indicate the decrease in use of calcium-based white paint in combination with mineral-based black and red paint, necessitating the firing of vessels twice at different temperatures [117]. The shift towards monochrome painting in the entire WTC corresponds to increasing settlement size [108,109,118–120], which fits the expectations of our model regarding standardization as an outcome of increasing production. It should be noted that the WTC example also demonstrates different factors simultaneously affecting the diversity at different levels of taxonomic hierarchy. Therefore, conditional diversities in ‘complex traits’ and their impact on the joint diversity at different scales [16,121,122], from an individual object to sets of objects, is a prominent topic for future research.

## Conclusion

Material signals reflecting social dynamics can be extracted from a great variety of archaeological time series, most notably ceramics. However, the results presented here dampen expectations to easily base reconstructions of social dynamics on single proxies, as analyses of possible impacts of social complexity on the diversity of archaeological data require an integration of population proxies and data on the organization of production. Moreover, it is important to have at least a coarse understanding of the coding system and what content the signals once communicated, to infer social dynamics. Lastly, path-dependency, the impact of stochastic processes, and equifinality of production and cultural transmission biases need to be considered as well. This broadening of factors leads to a broadening of possible trajectories: apart from an increase in diversity, standardization (a decrease in variation) may be an equally viable signal of increasing social complexity. In fact, as our mathematical simulation indicates, both trajectories are related to each other (Fig 1a). Therefore, future studies will focus on the further elaboration of the above-presented model in order to understand how scale shifts towards larger social entities are interrelated with standardization processes.

## Supporting information

S1 File All data underlying Figs 2–10 is available from the supporting information Table S1. (XLSX)
