# Supplementary material for: Social dynamics signals in archaeological time-series: How cultural transmission and production biases shape assemblage diversity
Source: PLoS One. 2026 Oct 7;21(10):e0358135. doi: 10.1371/journal.pone.0358135 (PMC13645070; doi:10.1371/journal.pone.0358135)
Supplement: Supplementary file 1 [file pone.0358135.s002.docx]

The authors present an algorithmic model for assessing change (as measuring by diversity) in archaeological assemblages – here in prehistoric European Neolithic pottery traditions. They do so as a way of querying changing social cycles in the human past as those cycles are reflected in the material record. They discuss this in relation to transmission processes – particularly transmission/production biases.

It is welcome to see that from the outset, the authors astutely note that both positive and negative (or no) correlations between social complexity and material diversity may potentially reflect similar processes (e.g., demography) driving change.

Lines 54-55: For sentence flow, change “Occasionally, may social cycles trajectories….”

To ‘Occasionally, social cycle trajectories may….’

Line 73: move the word “were” to read “…based on time series of ceramics, Gronenborn et al. were able to….”

I am not sure that I agree that (Lines 98-99) “adoption of cultural traits from other groups” is best described as ‘mutation’ but I understand where the authors are going with this. I would suggest that mutation in the sense of material culture is more a product of accidental invention and subsequent successful transmission of the new trait. The authors actually touch on this correctly in line 456. But perhaps this is nit-picky of me? Either way, they are correct in concluding that both drift and mutation shape neutral transmission.

Lines 129-131: I think this observation makes more sense if reversed, given the points made in the above paragraph, no? Because the material culture is not necessarily altering the complexity of the society, but rather the complexity of the society is being reflected in changes in the material culture. Perhaps I am being pedantic?

“From this, it follows that changes in material culture diversity are inherently linked to changes in social complexity.”

Lines 268-270: The authors state “A lower proportion of the primary unit presumes a higher number of the secondary units and higher diversity at each taxonomic level.” But why? Is this simply a feature of the model assumptions or is there a real-world logic to these assumptions? Perhaps a sentence clarifying this may be in order?

Line 362: Should “short-time” be changed to ‘short-term’?

Lines 452-453: This sentence does not make sense as written or is merely incomplete.

Line 526: add a comma after ‘houses’

Line 586: move the word “need” to after the word “biases” on line 587

Line 591: move the word “are” to after “trajectories”

Line 591: move the word “will” to after “studies”

**General Comments**

As the authors’ simulation specifically focuses on changes in style traits rather than functional units, it may be fitting to add a sentence or two discussing Robert Dunnell’s (1978) insights into this very phenomenon. Specifically, Dunnell’s (1978: 199) assertion that “Style denotes those forms that do not have detectable selective values. Function is manifest as those forms that directly affect the Darwinian fitness of the populations in which they occur.” But we know that ‘style’ is indeed often selected for by people for various reasons, which I think is important for the authors’ overall point about build up and reverse of diversity based on what may arguably be a neutral or nearly-neutral suite of traits (at least hypothetically). This also relates to transmission bias – particularly *model-based bias* or *frequency-based bias* when individuals over time (and in a growing population) are exposed to aesthetic and/or culturally valued iconography or symbolism, etc. I gather the authors are considering these points in pages 18-20 (and e.g., lines 460-471 and lines 501-503) but it could perhaps be made more explicit.

The authors’ may benefit from acknowledging that a major aspect of their assumptions is that producers and consumers are different individuals (which makes sense given the LBK case contexts). While is small-scale societies this may often not be the case. I get that this is not the subject of this work, but it may be helpful to establish recognition of this fact and how it relates to the model as set forth?

**Conclusion**

Overall, the paper is well written and the content is presented in an understandable manner. The proposed model is presented clearly and demonstrated/simulated convincingly – including the caveat that the phenomena being modeled and the signals produced are complex and should be approached with an interpretive level of caution. But such is good science. I will admit that I did not attempt my own tests of the formula(s) or broader model and got a bit lost in the section on ‘*Total production and the maximal proportion of a trait*’. This said, am I correct in gathering that Equations 6-7 in essence allow for accounting for the process of cultural ‘ratcheting’? If I follow the equations correctly, this seems an elegant way of so doing.

**Suggestion**

This paper should be published with minimal changes/edits such as those given above. The paper is a valuable contribution to the algorithmic modeling of processes of culture change in relation to material culture production.

Thank you for the opportunity to review this work.
