# Supplementary material for: Social dynamics signals in archaeological time-series: How cultural transmission and production biases shape assemblage diversity
Source: PLoS One. 2026 Oct 7;21(10):e0358135. doi: 10.1371/journal.pone.0358135 (PMC13645070; doi:10.1371/journal.pone.0358135)
Supplement: Supplementary file 2 [file pone.0358135.s004.docx]

**Response to the Editor’s and Reviewers’ Comments**

**Editor’s comments**

**Editor’s comment**: Please ensure that your manuscript meets PLOS ONE's style requirements, including those for file naming. The PLOS ONE style templates can be found at
https://journals.plos.org/plosone/s/file?id=wjVg/PLOSOne_formatting_sample_main_body.pdf and
<https://journals.plos.org/plosone/s/file?id=ba62/PLOSOne_formatting_sample_title_authors_affiliations.pdf>

**Reply**: We made the necessary to changes to the manuscript to meet PLOS ONE’s style requirements

**Editor’s comment**: Please note that PLOS One has specific guidelines on code sharing for submissions in which author-generated code underpins the findings in the manuscript. In these cases, we expect all author-generated code to be made available without restrictions upon publication of the work. Please review our guidelines at <https://journals.plos.org/plosone/s/materials-and-software-sharing#loc-sharing-code> and ensure that your code is shared in a way that follows best practice and facilitates reproducibility and reuse.

**Reply**: We did not generate any code.

**Editor’s comment**: We noted in your submission details that a portion of your manuscript may have been presented or published elsewhere. Please clarify whether this publication was peer-reviewed and formally published. If this work was previously peer-reviewed and published, in the cover letter please provide the reason that this work does not constitute dual publication and should be included in the current manuscript.

**Reply**: In order to explain our line of argument it was necessary to refer to previous publications. These are just briefly introduced. The respective figures were adapted and changed specifically for the PloS publication. We also included references to a paper, which was published in early 2026. We had not cited this paper previously due to the PLoS’ restrictions on citing unpublished work. In the cover letter, we included detailed explanation for the reasons that this work does not constitute dual publication and why the reference should be included in the current manuscript.

**Editor’s comment**: We note that Figures 7 and 10 in your submission contain copyrighted images. All PLOS content is published under the Creative Commons Attribution License (CC BY 4.0), which means that the manuscript, images, and Supporting Information files will be freely available online, and any third party is permitted to access, download, copy, distribute, and use these materials in any way, even commercially, with proper attribution. For more information, see our copyright guidelines: <http://journals.plos.org/plosone/s/licenses-and-copyright>.

We require you to either (1) present written permission from the copyright holder to publish these figures specifically under the CC BY 4.0 license, or (2) remove the figures from your submission:

**Reply**: We changed Figs 7 and 10. Fig 7 now shows two new vessels from a German publication. The filled-out copyright transfer form is included. Fig 10 now includes the image of two WTC vessels, adopted from Ryzhov et al. 2021 (<https://journals.ich.md/index.php/archaeology/en/issue/view/32/31>), which is published under the Creative Commons Attribution License (CC BY 4.0: see https://journals.ich.md/index.php/archaeology/en/about-the-journal) and, therefore, does not require permission.

**Editor’s comment**: If the reviewer comments include a recommendation to cite specific previously published works, please review and evaluate these publications to determine whether they are relevant and should be cited. There is no requirement to cite these works unless the editor has indicated otherwise.

**Reply**: We accepted the Reviewer’s recommendation to cite R. Dunnell’s work and included several more citations accompanying the entries, which follow Reviewers’ comments.

**Editor’s comment**: Please review your reference list to ensure that it is complete and correct. If you have cited papers that have been retracted, please include the rationale for doing so in the manuscript text, or remove these references and replace them with relevant current references. Any changes to the reference list should be mentioned in the rebuttal letter that accompanies your revised manuscript. If you need to cite a retracted article, indicate the article’s retracted status in the References list and also include a citation and full reference for the retraction notice.

**Reply**: we reviewed the reference list and ensured that it is complete and correct. Changes to the reference list are mentioned in the Cover Letter.

**Reviewers’ comments**

Reviewer #1

**Reviewer’s comment**: The authors present an algorithmic model for assessing change (as measuring by diversity) in archaeological assemblages – here in prehistoric European Neolithic pottery traditions. They do so as a way of querying changing social cycles in the human past as those cycles are reflected in the material record. They discuss this in relation to transmission processes – particularly transmission/production biases.

It is welcome to see that from the outset, the authors astutely note that both positive and negative (or no) correlations between social complexity and material diversity may potentially reflect similar processes (e.g., demography) driving change.

**Reply**: we are grateful to the Reviewer for the careful consideration of our paper and valuable comments and suggestions

**Reviewer’s comment**: Lines 54-55: For sentence flow, change “Occasionally, may social cycles trajectories….” To ‘Occasionally, social cycle trajectories may….’

**Reply**: corrected

**Reviewer’s comment:** Line 73: move the word “were” to read “…based on time series of ceramics, Gronenborn et al. were able to….”

**Reply**: corrected

**Reviewer’s comment:** I am not sure that I agree that (Lines 98-99) “adoption of cultural traits from other groups” is best described as ‘mutation’ but I understand where the authors are going with this. I would suggest that mutation in the sense of material culture is more a product of accidental invention and subsequent successful transmission of the new trait. The authors actually touch on this correctly in line 456. But perhaps this is nit-picky of me? Either way, they are correct in concluding that both drift and mutation shape neutral transmission.

**Reply**: here, we followed Neiman’s (1995) seminal paper and follow-up studies. Neiman specifically outs innovation (‘cultural mutation’) as follows: “I now want to introduce a second set of forces into our model, ones that introduce novel, selectively neutral variation into the population. These forces include both *in situ innovation* that arises within a group and *innovation that may be introduced into it from outside*”. We corrected the according sentence in our text as follows: “Diversity of the stylistic traits in the assemblage could be caused by cultural drift (random events) and/or ‘mutation’ (local innovation or adoption of cultural traits from other groups), both of which shape neutral cultural transmission [14, 45-48]” (lines 102-103; hereinafter we list line numbers from the ‘Revised Article with Changes Highlighted’)

**Reviewer’s comment:** Lines 129-131: I think this observation makes more sense if reversed, given the points made in the above paragraph, no? Because the material culture is not necessarily altering the complexity of the society, but rather the complexity of the society is being reflected in changes in the material culture. Perhaps I am being pedantic?

“From this, it follows that changes in material culture diversity are inherently linked to changes in social complexity.”

**Reply**: Good point! We corrected the text as suggested by the Reviewer.

**Reviewer’s comment:** Lines 268-270: The authors state “A lower proportion of the primary unit presumes a higher number of the secondary units and higher diversity at each taxonomic level.” But why? Is this simply a feature of the model assumptions or is there a real-world logic to these assumptions? Perhaps a sentence clarifying this may be in order?

**Reply**: A lower proportion of the primary unit presuming a higher number of the secondary units comes from our definition of the primary unit, which proportion exceeds the proportion of the secondary units. Increase in entropy (and diversity as the exponentiated entropy) with the increasing number of aggregation units is the property of entropy. We included the according reference to Gromov’s paper discussing properties of entropy and extended this part of the text as follows: “Since diversity increases with increasing number of units [68], a lower proportion of the primary unit presumes a higher number of the secondary units and higher diversity” (Lines 285-286)

**Reviewer’s comment:** Line 362: Should “short-time” be changed to ‘short-term’?

**Reply**: corrected

**Reviewer’s comment:** Lines 452-453: This sentence does not make sense as written or is merely incomplete.

**Reply**: We clarified this statement putting it as follows: “Equilibrium production results from the unchanged proportion of producers and consumers. While this equilibrium (production stability) holds, the innovation and cultural transmission biases are the only factors of material culture change (Table 1).” (Lines 475-477)

**Reviewer’s comment:** Line 526: add a comma after ‘houses’

**Reply**: corrected

**Reviewer’s comment:** Line 586: move the word “need” to after the word “biases” on line 587

**Reply**: corrected

**Reviewer’s comment:** Line 591: move the word “are” to after “trajectories”

**Reply**: corrected

**Reviewer’s comment:** Line 591: move the word “will” to after “studies”

**Reply**: corrected

**Reviewer’s comment:** General Comments
As the authors’ simulation specifically focuses on changes in style traits rather than functional units, it may be fitting to add a sentence or two discussing Robert Dunnell’s (1978) insights into this very phenomenon. Specifically, Dunnell’s (1978: 199) assertion that “Style denotes those forms that do not have detectable selective values. Function is manifest as those forms that directly affect the Darwinian fitness of the populations in which they occur.” But we know that ‘style’ is indeed often selected for by people for various reasons, which I think is important for the authors’ overall point about build up and reverse of diversity based on what may arguably be a neutral or nearly-neutral suite of traits (at least hypothetically). This also relates to transmission bias – particularly model-based bias or frequency-based bias when individuals over time (and in a growing population) are exposed to aesthetic and/or culturally valued iconography or symbolism, etc. I gather the authors are considering these points in pages 18-20 (and e.g., lines 460-471 and lines 501-503) but it could perhaps be made more explicit.

**Reply**: we referenced Dunnell’s paper including the definitions of style and function (Lines 137-140). We also included a short explanation of one of our examples to be more explicit regarding the style: “replacement of several forms **referring to the style** along with the introduction and extinction of several vessel types is visible as a rise-and-fall pattern in assemblage diversity c. 4100-3600 BCE” (Lines 98-101). We also agree that we should more explicitly address that individuals over time (also accounting for growing population and production bias) are exposed to aesthetic and/or culturally valued iconography or symbolism, etc. Therefore, we explained how the mathematical component of our model captures this as follows: “Any trait may gain or abandon its selective value over time. Unbiased transmission presumes that $p_{{{\alpha_{m}}_{\lambda}}_{t}}\approx p_{{{\alpha_{m}}_{\lambda}}_{t+1}}$ [49]. Therefore, according to Equation 4, replication rate ${r_{\alpha}}_{m_{\lambda}}$, which is equal to or approaching $r_{\alpha_{stab}}$, is indicative for a style, whereas replication rate ${r_{\alpha}}_{m_{\lambda}}$, which is sufficiently lower or higher than $r_{\alpha_{stab}}$, is indicative for a function (frequency-dependent bias). Note that, according to Equation 5, proportion and replication rate of the secondary units, ${r_{n}}_{m_{\lambda}}$ are also conditioned by the values of ${r_{\alpha}}_{m_{\lambda}}$.” (Lines 263-268).

**Reviewer’s comment:** The authors’ may benefit from acknowledging that a major aspect of their assumptions is that producers and consumers are different individuals (which makes sense given the LBK case contexts). While is small-scale societies this may often not be the case. I get that this is not the subject of this work, but it may be helpful to establish recognition of this fact and how it relates to the model as set forth?

**Reply**: In earlier version of this manuscript, we already stated that “… producers are most frequently also the consumers of their own products”. Agreeing with the Reviewer that this often the case in small-scale societies, we included this as follows: “… and considers that producers are most frequently also the consumers of their own products, **especially in small-scale societies**.” (Lines 139-140)

**Reviewer’s comment:** Conclusion
Overall, the paper is well written and the content is presented in an understandable manner. The proposed model is presented clearly and demonstrated/simulated convincingly – including the caveat that the phenomena being modeled and the signals produced are complex and should be approached with an interpretive level of caution. But such is good science. I will admit that I did not attempt my own tests of the formula(s) or broader model and got a bit lost in the section on ‘Total production and the maximal proportion of a trait’. This said, am I correct in gathering that Equations 6-7 in essence allow for accounting for the process of cultural ‘ratcheting’? If I follow the equations correctly, this seems an elegant way of so doing.

**Reply**: Yes, the Reviewer is correct regarding the Ratchet effect. This model’s behavior is accounted by Equation 4, and is specifically controlled by Equations 6-7. We added the explanation according to the broader Equation 4 (Lines 243-247).

**Reviewer’s comment:** Suggestion
This paper should be published with minimal changes/edits such as those given above. The paper is a valuable contribution to the algorithmic modeling of processes of culture change in relation to material culture production.

Thank you for the opportunity to review this work.

**Reply**: We are grateful to the Reviewer for a high evaluation of our contribution and the valuable comments and suggestions

Reviewer #2: Review of Diachenko & Gronenborn, Social Dynamics
Michael Kempf, Cambridge

I prefer an open and transparent review process, hence I disclose my identity.

**Reviewer’s comment:** The article provides a mathematical formulation of social complexity expressed in decorative stylistic features of archaeological assemblages. The authors develop an algorithm to evaluate social diversity vs uniformity in a simple model of production bias.
The paper is well written and offers a fresh perspective on the traditional and more theoretical relationship of pottery decoration and social complexity. The authors provide a comprehensive overview and, building on their long-standing experience in theoretical archaeology and particularly quantitative and computational approaches in the field, I do not see an argument against publication of this paper.
I also see the potential to update the model and increase its complexity (though this counteracts the model theory) including different variables, e.g., environmental contributors to socio-cultural diversifications (see chaotic events) and extreme periods as drivers of diversification or uniformity, e.g., via increased mobility patterns.

**Reply**: we are grateful to Michael Kempf for careful consideration of our research, valuable comments and suggestions, as well as recognizing the potential for further development of our model.

**Reviewer’s comment:** Minor issues are probably language related, e.g. the use of “Lets us start” (see, for example, line 264, 281, 376 but also other occasions). Id recommend using a more general term or delete the phrase.

**Reply**: corrected

**Reviewer’s comment:** Some formulations and comma placements sound a bit odd (e.g. Therefore, will future… (line 591, but also other occasions). I recommend to give the manuscript a fresh editing process and reformulate some of the rather German-sounding translations into English (e.g. line 273 following).

**Reply**: the language component of this paper was edited. We paraphrased several expressions and fixed a number of punctuation errors.

**Reviewer’s comment:** The figures are allright but could benefit from some stylistic polishing. I recommend reducing the color codes to a more “friendly” colorramp, e.g. Figs. 9 and 8, where colorblind people would probably not be able to differentiate the types mentioned in the legend. The orange/green combination of the first figures is also not very appealing and could be revised. Given the simplicity of Figs. 1-3, I wonder if these could be combined into a panel figure rather than stand-alone? I just imagine that in print this would look a bit too simple, but that is my personal opinion.

**Reply**: Figs 2-10 were reproduced using the Okabe–Ito color palette, which was specifically designed to be accessible to individuals with color vision deficiencies. For Fig 8, the Okabe–Ito palette was supplemented with additional high-contrast colors to ensure clear visual distinction. We agree that Figs 1-3 are quite simple, but they illustrate quite different portions of the text. These are the assumed processes behind assemblage diversity (Fig 1), general properties of entropy (Fig 2), and threshold controlling model’s behavior (Fig 3). Therefore, we prefer remaining these three images as three different figures.
