# Supplementary material for: Social dynamics signals in archaeological time-series: How cultural transmission and production biases shape assemblage diversity
Source: PLoS One. 2026 Oct 7;21(10):e0358135. doi: 10.1371/journal.pone.0358135 (PMC13645070; doi:10.1371/journal.pone.0358135)
Supplement: Peer review history

# Peer review: Decision Letter 0

**DOI:** 10.1371/journal.pone.0358135.r001

**Authors:** Raven Garvey

15 Jun 2026

Dear Dr. Gronenborn,

Thank you for submitting your manuscript to PLOS ONE. After careful consideration, we feel that it has merit but does not fully meet PLOS ONE’s publication criteria as it currently stands. Therefore, we invite you to submit a revised version of the manuscript that addresses the points raised during the review process.

As you address the reviewers' comments, we would like for you to attend in particular to those requesting clarification.

Please submit your revised manuscript by Jul 30 2026 11:59PM. If you will need more time than this to complete your revisions, please reply to this message or contact the journal office at plosone@plos.org. When you're ready to submit your revision, log on to https://www.editorialmanager.com/pone/ and select the 'Submissions Needing Revision' folder to locate your manuscript file.

- A letter that responds to each point raised by the academic editor and reviewer(s). You should upload this letter as a separate file labeled 'Response to Reviewers'.
- A marked-up copy of your manuscript that highlights changes made to the original version. You should upload this as a separate file labeled 'Revised Manuscript with Track Changes'.
- An unmarked version of your revised paper without tracked changes. You should upload this as a separate file labeled 'Manuscript'.

If you would like to make changes to your financial disclosure, please include your updated statement in your cover letter. Guidelines for resubmitting your figure files are available below the reviewer comments at the end of this letter.

If applicable, we recommend that you deposit your laboratory protocols in protocols.io to enhance the reproducibility of your results. Protocols.io assigns your protocol its own identifier (DOI) so that it can be cited independently in the future. For instructions see: https://journals.plos.org/plosone/s/submission-guidelines#loc-laboratory-protocols. Additionally, PLOS ONE offers an option for publishing peer-reviewed Lab Protocol articles, which describe protocols hosted on protocols.io. Read more information on sharing protocols at https://plos.org/protocols?utm_medium=editorial-email&utm_source=authorletters&utm_campaign=protocols.

As the corresponding author, your ORCID iD is verified in the submission system and will appear in the published article. PLOS supports the use of ORCID, and we encourage all coauthors to register for an ORCID iD and use it as well. Please encourage your coauthors to verify their ORCID iD within the submission system before final acceptance, as unverified ORCID iDs will not appear in the published article. *Only*  the individual author can complete the verification step; PLOS staff *cannot*  verify ORCID iDs on behalf of authors.

We look forward to receiving your revised manuscript.

Kind regards,

Raven Garvey, Ph.D.

Academic Editor

PLOS One

Journal Requirements:

When submitting your revision, we need you to address these additional requirements.

1. Please ensure that your manuscript meets PLOS ONE's style requirements, including those for file naming. The PLOS ONE style templates can be found at

https://journals.plos.org/plosone/s/file?id=wjVg/PLOSOne_formatting_sample_main_body.pdf and

https://journals.plos.org/plosone/s/file?id=ba62/PLOSOne_formatting_sample_title_authors_affiliations.pdf

2. Please note that PLOS One has specific guidelines on code sharing for submissions in which author-generated code underpins the findings in the manuscript. In these cases, we expect all author-generated code to be made available without restrictions upon publication of the work. Please review our guidelines at https://journals.plos.org/plosone/s/materials-and-software-sharing#loc-sharing-code and ensure that your code is shared in a way that follows best practice and facilitates reproducibility and reuse.

3. We noted in your submission details that a portion of your manuscript may have been presented or published elsewhere. Please clarify whether this publication was peer-reviewed and formally published. If this work was previously peer-reviewed and published, in the cover letter please provide the reason that this work does not constitute dual publication and should be included in the current manuscript.

4. We note that Figures 7 and 10 in your submission contain copyrighted images. All PLOS content is published under the Creative Commons Attribution License (CC BY 4.0), which means that the manuscript, images, and Supporting Information files will be freely available online, and any third party is permitted to access, download, copy, distribute, and use these materials in any way, even commercially, with proper attribution. For more information, see our copyright guidelines: http://journals.plos.org/plosone/s/licenses-and-copyright.

We require you to either (1) present written permission from the copyright holder to publish these figures specifically under the CC BY 4.0 license, or (2) remove the figures from your submission:

a. You may seek permission from the original copyright holder of Figures 7 and 10 to publish the content specifically under the CC BY 4.0 license.

We recommend that you contact the original copyright holder with the Content Permission Form (http://journals.plos.org/plosone/s/file?id=7c09/content-permission-form.pdf) and the following text:

“I request permission for the open-access journal PLOS ONE to publish XXX under the Creative Commons Attribution License (CCAL) CC BY 4.0 (http://creativecommons.org/licenses/by/4.0/). Please be aware that this license allows unrestricted use and distribution, even commercially, by third parties. Please reply and provide explicit written permission to publish XXX under a CC BY license and complete the attached form.”

Please upload the completed Content Permission Form or other proof of granted permissions as an "Other" file with your submission.

In the figure caption of the copyrighted figure, please include the following text: “Reprinted from [ref] under a CC BY license, with permission from [name of publisher], original copyright [original copyright year].”

b. If you are unable to obtain permission from the original copyright holder to publish these figures under the CC BY 4.0 license or if the copyright holder’s requirements are incompatible with the CC BY 4.0 license, please either i) remove the figure or ii) supply a replacement figure that complies with the CC BY 4.0 license. Please check copyright information on all replacement figures and update the figure caption with source information. If applicable, please specify in the figure caption text when a figure is similar but not identical to the original image and is therefore for illustrative purposes only.

5. If the reviewer comments include a recommendation to cite specific previously published works, please review and evaluate these publications to determine whether they are relevant and should be cited. There is no requirement to cite these works unless the editor has indicated otherwise.

6. Please review your reference list to ensure that it is complete and correct. If you have cited papers that have been retracted, please include the rationale for doing so in the manuscript text, or remove these references and replace them with relevant current references. Any changes to the reference list should be mentioned in the rebuttal letter that accompanies your revised manuscript. If you need to cite a retracted article, indicate the article’s retracted status in the References list and also include a citation and full reference for the retraction notice.

[Note: HTML markup is below. Please do not edit.]

Reviewers' comments:

Reviewer's Responses to Questions

**Comments to the Author**

1. Is the manuscript technically sound, and do the data support the conclusions?

Reviewer #1: Yes

Reviewer #2: Yes

**********

2. Has the statistical analysis been performed appropriately and rigorously?

Reviewer #1: I Don't Know

Reviewer #2: Yes

**********

3. Have the authors made all data underlying the findings in their manuscript fully available?

The PLOS Data policy

Reviewer #1: Yes

Reviewer #2: Yes

**********

4. Is the manuscript presented in an intelligible fashion and written in standard English?

Reviewer #1: Yes

Reviewer #2: Yes

**********

Reviewer #1: The authors present an algorithmic model for assessing change (as measuring by diversity) in archaeological assemblages – here in prehistoric European Neolithic pottery traditions. They do so as a way of querying changing social cycles in the human past as those cycles are reflected in the material record. They discuss this in relation to transmission processes – particularly transmission/production biases.

It is welcome to see that from the outset, the authors astutely note that both positive and negative (or no) correlations between social complexity and material diversity may potentially reflect similar processes (e.g., demography) driving change.

Lines 54-55: For sentence flow, change “Occasionally, may social cycles trajectories….”

To ‘Occasionally, social cycle trajectories may….’

Line 73: move the word “were” to read “…based on time series of ceramics, Gronenborn et al. were able to….”

I am not sure that I agree that (Lines 98-99) “adoption of cultural traits from other groups” is best described as ‘mutation’ but I understand where the authors are going with this. I would suggest that mutation in the sense of material culture is more a product of accidental invention and subsequent successful transmission of the new trait. The authors actually touch on this correctly in line 456. But perhaps this is nit-picky of me? Either way, they are correct in concluding that both drift and mutation shape neutral transmission.

Lines 129-131: I think this observation makes more sense if reversed, given the points made in the above paragraph, no? Because the material culture is not necessarily altering the complexity of the society, but rather the complexity of the society is being reflected in changes in the material culture. Perhaps I am being pedantic?

“From this, it follows that changes in material culture diversity are inherently linked to changes in social complexity.”

Lines 268-270: The authors state “A lower proportion of the primary unit presumes a higher number of the secondary units and higher diversity at each taxonomic level.” But why? Is this simply a feature of the model assumptions or is there a real-world logic to these assumptions? Perhaps a sentence clarifying this may be in order?

Line 362: Should “short-time” be changed to ‘short-term’?

Lines 452-453: This sentence does not make sense as written or is merely incomplete.

Line 526: add a comma after ‘houses’

Line 586: move the word “need” to after the word “biases” on line 587

Line 591: move the word “are” to after “trajectories”

Line 591: move the word “will” to after “studies”

General Comments

As the authors’ simulation specifically focuses on changes in style traits rather than functional units, it may be fitting to add a sentence or two discussing Robert Dunnell’s (1978) insights into this very phenomenon. Specifically, Dunnell’s (1978: 199) assertion that “Style denotes those forms that do not have detectable selective values. Function is manifest as those forms that directly affect the Darwinian fitness of the populations in which they occur.” But we know that ‘style’ is indeed often selected for by people for various reasons, which I think is important for the authors’ overall point about build up and reverse of diversity based on what may arguably be a neutral or nearly-neutral suite of traits (at least hypothetically). This also relates to transmission bias – particularly model-based bias or frequency-based bias when individuals over time (and in a growing population) are exposed to aesthetic and/or culturally valued iconography or symbolism, etc. I gather the authors are considering these points in pages 18-20 (and e.g., lines 460-471 and lines 501-503) but it could perhaps be made more explicit.

The authors’ may benefit from acknowledging that a major aspect of their assumptions is that producers and consumers are different individuals (which makes sense given the LBK case contexts). While is small-scale societies this may often not be the case. I get that this is not the subject of this work, but it may be helpful to establish recognition of this fact and how it relates to the model as set forth?

Conclusion

Overall, the paper is well written and the content is presented in an understandable manner. The proposed model is presented clearly and demonstrated/simulated convincingly – including the caveat that the phenomena being modeled and the signals produced are complex and should be approached with an interpretive level of caution. But such is good science. I will admit that I did not attempt my own tests of the formula(s) or broader model and got a bit lost in the section on ‘Total production and the maximal proportion of a trait’. This said, am I correct in gathering that Equations 6-7 in essence allow for accounting for the process of cultural ‘ratcheting’? If I follow the equations correctly, this seems an elegant way of so doing.

Suggestion

This paper should be published with minimal changes/edits such as those given above. The paper is a valuable contribution to the algorithmic modeling of processes of culture change in relation to material culture production.

Thank you for the opportunity to review this work.

Reviewer #2: Review of Diachenko & Gronenborn, Social Dynamics

Michael Kempf, Cambridge

I prefer an open and transparent review process, hence I disclose my identity.

The article provides a mathematical formulation of social complexity expressed in decorative stylistic features of archaeological assemblages. The authors develop an algorithm to evaluate social diversity vs uniformity in a simple model of production bias.

The paper is well written and offers a fresh perspective on the traditional and more theoretical relationship of pottery decoration and social complexity. The authors provide a comprehensive overview and, building on their long-standing experience in theoretical archaeology and particularly quantitative and computational approaches in the field, I do not see an argument against publication of this paper.

I also see the potential to update the model and increase its complexity (though this counteracts the model theory) including different variables, e.g., environmental contributors to socio-cultural diversifications (see chaotic events) and extreme periods as drivers of diversification or uniformity, e.g., via increased mobility patterns.

Minor issues are probably language related, e.g. the use of “Lets us start” (see, for example, line 264, 281, 376 but also other occasions). Id recommend using a more general term or delete the phrase.

Some formulations and comma placements sound a bit odd (e.g. Therefore, will future… (line 591, but also other occasions). I recommend to give the manuscript a fresh editing process and reformulate some of the rather German-sounding translations into English (e.g. line 273 following).

The figures are allright but could benefit from some stylistic polishing. I recommend reducing the color codes to a more “friendly” colorramp, e.g. Figs. 9 and 8, where colorblind people would probably not be able to differentiate the types mentioned in the legend. The orange/green combination of the first figures is also not very appealing and could be revised. Given the simplicity of Figs. 1-3, I wonder if these could be combined into a panel figure rather than stand-alone? I just imagine that in print this would look a bit too simple, but that is my personal opinion.

**********

what does this mean?). If published, this will include your full peer review and any attached files.

If you choose “no”, your identity will remain anonymous but your review may still be made public.

**Do you want your identity to be public for this peer review?**  For information about this choice, including consent withdrawal, please see our Privacy Policy

Reviewer #1: No

Reviewer #2: **Yes:** Michael Kempf

**********

[NOTE: If reviewer comments were submitted as an attachment file, they will be attached to this email and accessible via the submission site. Please log into your account, locate the manuscript record, and check for the action link "View Attachments". If this link does not appear, there are no attachment files.]

To ensure your figures meet our technical requirements, please review our figure guidelines: https://journals.plos.org/plosone/s/figures

You may also use PLOS’s free figure tool, NAAS, to help you prepare publication quality figures: https://journals.plos.org/plosone/s/figures#loc-tools-for-figure-preparation.

NAAS will assess whether your figures meet our technical requirements by comparing each figure against our figure specifications.

Attachment Submitted filename: PONE-D-25-57803_m1_review.docx

# Author comment: Author response to Decision Letter 1

**DOI:** 10.1371/journal.pone.0358135.r002

5 Aug 2026

We addressed all reviewer comments in the Cover Letter and Response to Editors' and Reviewers' Comments.

Attachment Submitted filename: Response to the Editors and Reviewers.docx

# Editor report: Decision Letter 1

**DOI:** 10.1371/journal.pone.0358135.r003

**Authors:** Raven Garvey

27 Aug 2026

Social dynamics signals in archaeological time-series: How cultural transmission and production biases shape assemblage diversity

PONE-D-25-57803R1

Dear Dr. Gronenborn,

We’re pleased to inform you that your manuscript has been judged scientifically suitable for publication and will be formally accepted for publication once it meets all outstanding technical requirements.

Within one week, you’ll receive an e-mail detailing the required amendments. When these have been addressed, you’ll receive a formal acceptance letter and your manuscript will be scheduled for publication.

An invoice will be generated when your article is formally accepted. Please note, if your institution has a publishing partnership with PLOS and your article meets the relevant criteria, all or part of your publication costs will be covered. Please make sure your user information is up-to-date by logging into Editorial Manager at Editorial Manager® and clicking the ‘Update My Information' link at the top of the page. For questions related to billing, please contact billing support.

If your institution or institutions have a press office, please notify them about your upcoming paper to help maximize its impact. If they’ll be preparing press materials, please inform our press team as soon as possible -- no later than 48 hours after receiving the formal acceptance. Your manuscript will remain under strict press embargo until 2 pm Eastern Time on the date of publication. For more information, please contact onepress@plos.org.

Kind regards,

Raven Garvey, Ph.D.

Academic Editor

PLOS One

Additional Editor Comments (optional):

Reviewers' comments:

# Editor report: Acceptance letter

**DOI:** 10.1371/journal.pone.0358135.r004

**Authors:** Raven Garvey

PONE-D-25-57803R1

PLOS One

Dear Dr. Gronenborn,

I'm pleased to inform you that your manuscript has been deemed suitable for publication in PLOS One. Congratulations! Your manuscript is now being handed over to our production team.

At this stage, our production department will prepare your paper for publication. This includes ensuring the following:

* All references, tables, and figures are properly cited

* All relevant supporting information is included in the manuscript submission,

* There are no issues that prevent the paper from being properly typeset

You will receive further instructions from the production team, including instructions on how to review your proof when it is ready. Please keep in mind that we are working through a large volume of accepted articles, so please give us a few days to review your paper and let you know the next and final steps.

Lastly, if your institution or institutions have a press office, please let them know about your upcoming paper now to help maximize its impact. If they'll be preparing press materials, please inform our press team within the next 48 hours. Your manuscript will remain under strict press embargo until 2 pm Eastern Time on the date of publication. For more information, please contact onepress@plos.org.

You will receive an invoice from PLOS for your publication fee after your manuscript has reached the completed accept phase. If you receive an email requesting payment before acceptance or for any other service, this may be a phishing scheme. Learn how to identify phishing emails and protect your accounts at https://explore.plos.org/phishing.

If we can help with anything else, please email us at customercare@plos.org.

Thank you for submitting your work to PLOS One and supporting open access.

Kind regards,

PLOS One Editorial Office Staff

on behalf of

Dr Raven Garvey

Academic Editor

PLOS One
